# Enantioselective 1,3-Dipolar Cycloaddition Using (*Z*)-α-Amidonitroalkenes as a Key Step to the Access to Chiral *cis*-3,4-Diaminopyrrolidines †

**DOI:** 10.3390/molecules27144579

**Published:** 2022-07-18

**Authors:** Eduardo García-Mingüens, Marcos Ferrándiz-Saperas, M. de Gracia Retamosa, Carmen Nájera, Miguel Yus, José M. Sansano

**Affiliations:** 1 Instituto de Síntesis Orgánica (ISO), Centro de Innovación en Química Avanzada (ORFEO-CINQA), Departamento de Química Orgánica, University of Alicante, P.O. Box 99, 03690 San Vicente del Raspeig, Spain; eduardo.gm@ua.es (E.G.-M.); marcos.ferrandiz@ua.es (M.F.-S.); gracia.retamosa@ua.es (M.d.G.R.); 2 Centro de Innovación en Química Avanzada (ORFEO-CINQA), P.O. Box 99, 03690 San Vicente del Raspeig, Spain; yus@ua.es

**Keywords:** nitroprolinates, enantioseletive cycloaddition, phosphoramidite, azomethine ylides, diamines

## Abstract

The enantioselective 1,3-dipolar cycloaddition between imino esters and (*Z*)-nitroalkenes bearing a masked amino group in the β-position was studied using several chiral ligands and silver salts. The optimized reaction conditions were directly applied to the study of the scope of the reaction. The determination of the absolute configuration was evaluated using NMR experiments and electronic circular dichroism (ECD). The reduction and hydrolysis of both groups was performed to generate in an excellent enantiomeric ratio the corresponding *cis-*2,3-diaminoprolinate.

## 1. Introduction

Chiral vicinal diamines are of tremendous interest to the synthetic chemist as they are found in many chiral catalysts (as chiral ligands or organocatalysts) and key intermediates in complex synthesis [1]. Currently, there is no unified approach to synthesize these chiral vicinal diamines, and they are often challenging to obtain, especially if unsymmetrically substituted. Recent studies dealt with the protonation of enamines [2], borylcopper-mediated homocouplins [3], diaza-Cope rearrangements [4], transformations of diols [5] and mefloquine [6], metal catalysis [7,8,9], Mannich reactions [10], nucleophilic trifluoromethylation [11,12], enantioselective reductive coupling of imines [13,14], *syn*-diamination of alkenes [15], etc.

**Figure 1:**
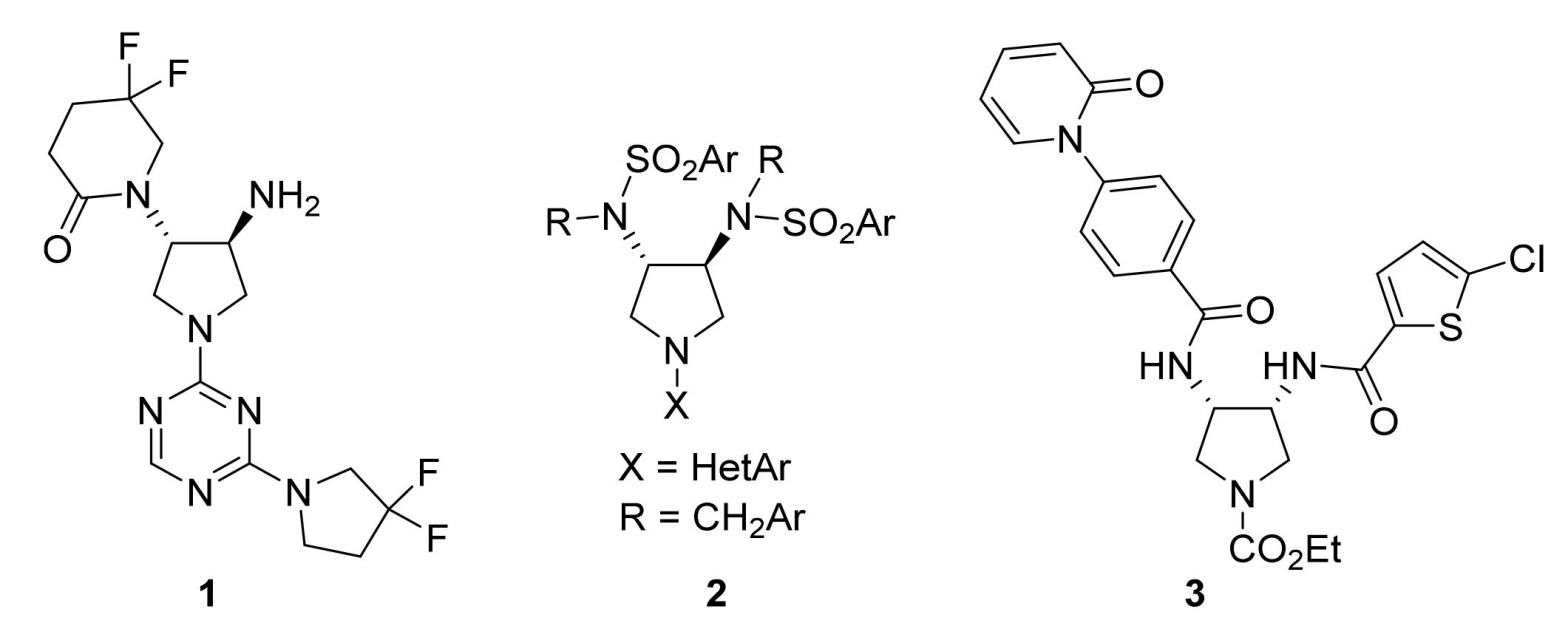
Bioactive structures incorporating a 3,4-diaminopyrrolidine unit.

In the particular example of enantiomerically enriched pyrrolidine-3,4-diamines, this skeleton is present in some biologically active compounds (Figure 1), but their synthesis is very complex, requiring many steps [16,17,18,19,20,21,22,23]. The *trans-*3,4-arrangement is key for the preparation of a dipeptidyl transferse-4 inhibitor **1** [16], HIV-1 protease inhibitors (type **2** structure) [17,18,19,20], human T cell leukemia virus-1 inhibitors (type **2** structure) [21], and antibiotics or antifungals (type **2** structure) [22]. The *cis*-configuration of the diamine is present in the non-symmetrical structure **3**, which is a very potent anticoagulant [23].

**Scheme 1:**
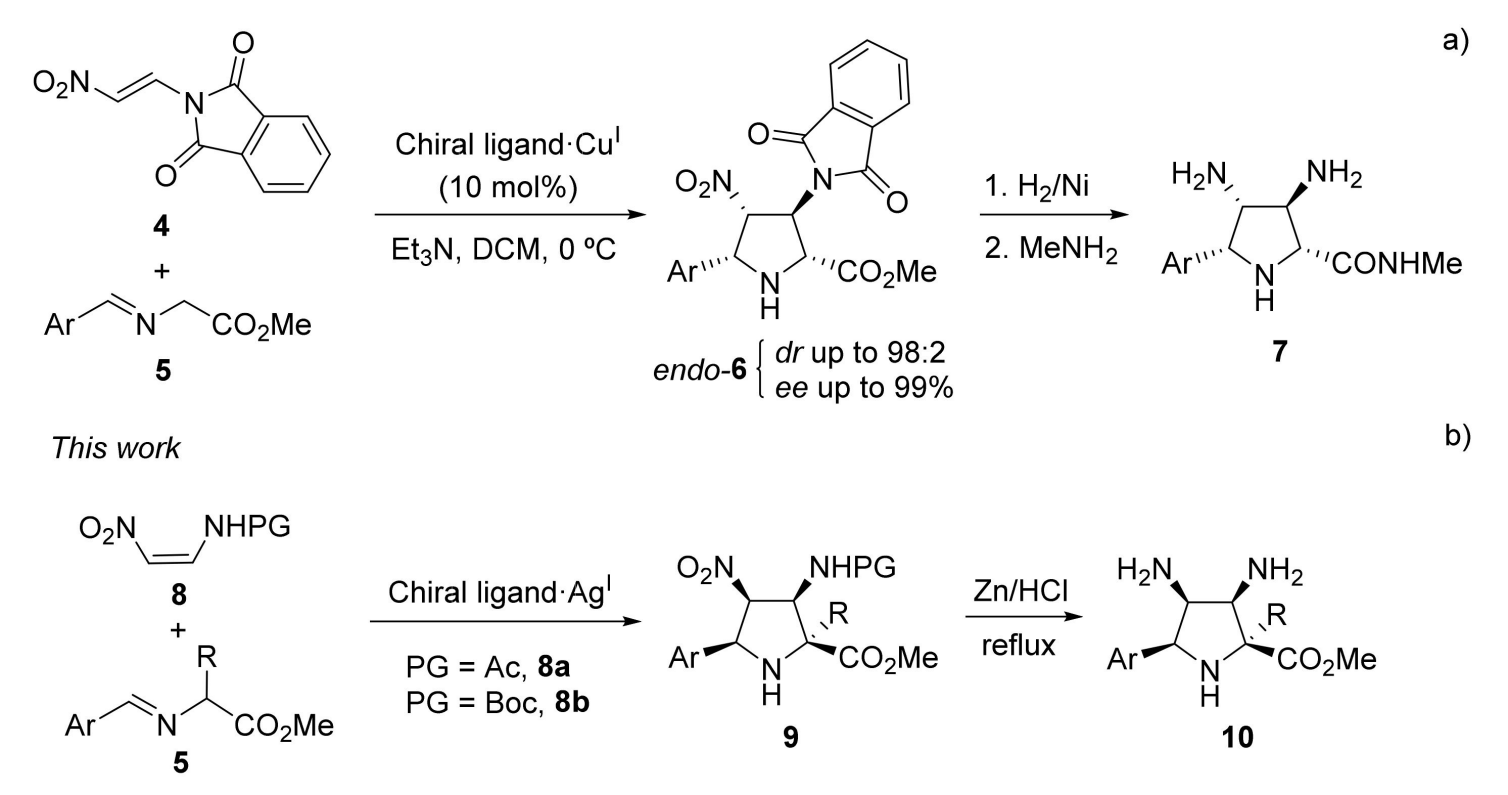
Enantioselective synthesis of 3,4-diaminopyrrolidines through 1,3-DC of stabilized azomethine ylides.

It is well known that the enantioselective 1,3-dipolar cycloaddition (1,3-DC) of fleeting azomethine ylides and alkenes rapidly gives access to pyrrolidines, with excellent results [24,25,26,27,28]. In this line, the selection of the appropriate dipolarophile can result in multiple functionalities at the 3- and 4-carbon atoms of this heterocycle. Thus, (*E*)-β-phthalimidonitroethene (**4**) [29,30,31,32,33,34,35,36,37,38,39,40,41] has been employed for the construction of the *trans*-3,4-diamino derivatives **7** using a chiral N,O-ligand/copper(I) complex [42] (Scheme 1a). However, the *cis*-relative configuration of 3,4-pyrrolidines **10** (extremely difficult to generate by other routes) in an enantioenriched form has not been reported yet. So, in this work, we first design the most appropriate nitroalkenes **8** to perform the classical chiral metal-catalyzed 1,3-DC from imino esters **5** (Scheme 1b).

## 2. Results and Discussion

**Scheme 2:**
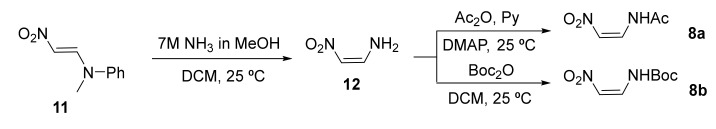
Synthesis of dipolarophiles **8a** and **8b**.

The initial design of the enantioselective 1,3-DC required the previous synthesis of *Z-*β-aminosubstituted nitroalkenes **8** using acetyl (**8a**)- or *tert-*butoxycarbonyl (**8b**)-protecting groups. Compound **8a** has been previously described [43] from the nitroalkene **11**. The intermediate nitroamine **12** was the direct precursor of **8a** and **8b** after the corresponding acylation in the presence of pyridine and *N,N*-dimethylaminopyridine at room temperature (25 °C, Scheme 2) [44]. However, compound **8b** has not been characterized yet.

**Scheme 3:**
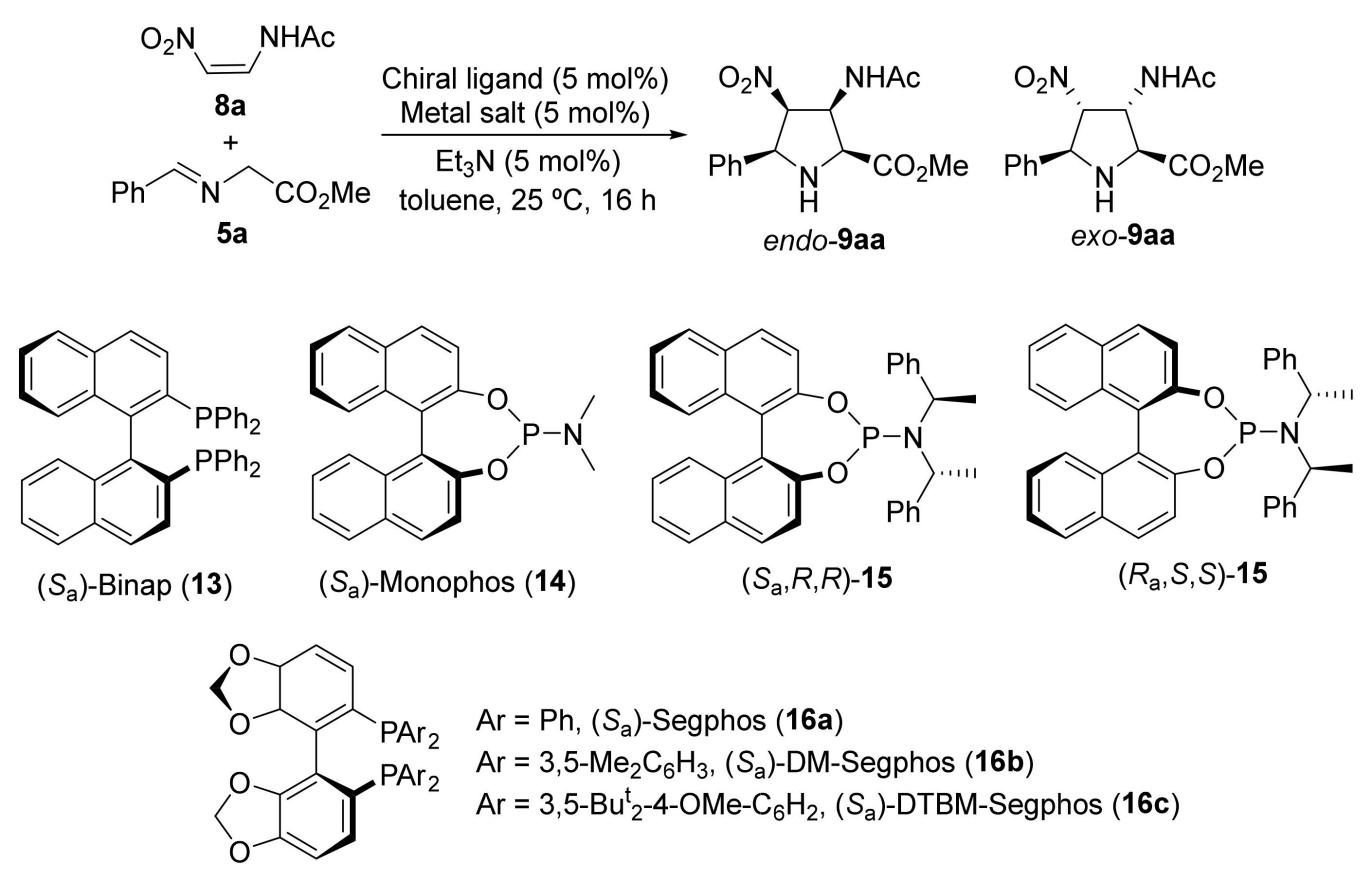
Optimization of the reaction between **5a** and **8a**.

According to the experience of our group related to the synthesis of enantiomerically enriched nitroprolinates [45,46,47,48,49], we employed a catalytic system formed by several ligands (5 mol%) and silver(I) or copper(I) salts (5 mol%). They were mixed in toluene and stirred for 30 min. After the generation of the catalytic active species, the imino ester **5a** and the dipolarophile **8a** were added. At the end, triethylamine (5 mol%) was added and the reaction was allowed to react for 16 h at room temperature (25 °C) (Scheme 3). This model reaction was studied, and the effects of several parameters are shown in Table 1. Several ligands as (*S*_a_)-Binap (**13**), (*S*_a_)-Monophos (**14**), (*S*_a_)-Segphos (**16a**), and its derivatives (*S*_a_)-**16b** and (*S*_a_)-**16c** were tested, offering very low chemical yields of the cycloadducts **9aa** (Table 1, entries 1, 2, and 4–6). However, phosphoramidite (*S*_a_,*R*,*R*)-**15** in combination with silver perchlorate afforded a high *endo-*diastereoselection (88:12), good chemical yield (77%), and excellent *ee* of the *endo-***9aa** cycloadduct (Table 1, entry 3). Copper(I) salts were not appropriate for this 1,3-DC, giving almost null conversions of the expected product (Table 1, entries 7–10). The match-mismatch combination of the two chiral environments present in ligand **15** was analyzed, finding that (*S*_a_,*R*,*R*)-**15** better furnished *dr* and *ee* than the (*S*_a_,*S*,*S*)-**15** phosphoramidite (Table 1, entry 11). The employment of the (*R*_a_,*S*,*S*)-**15** ligand afforded the opposite enantiomer in the same yield and diastereoselectivity (Table 1, entry 12). The screening of the silver salts was performed next (Table 1, entries 13–20). AgOTf and AgOAc afforded very close results of the major *endo**-*****9aa** compound but never improved the analogous one obtained in the reaction involving AgClO_4_ (Table 1, entries 13 and 17). In the example run with basic silver acetate, in the absence of triethylamine, the *ee* of *endo*-**9aa** was very low (Table 1, entry 14). Despite the higher *dr* (92:8) and identical *ee* obtained for *endo**-*****9aa**, the reaction performed at 0 °C occurred in a lower yield than the process run at room temperature (Table 1, entry 21). The employment of THF, acetonitrile, or DCM instead of toluene as solvent, or substitution of triethylamine by DABCO or diisopropylethylamine (not shown in Table 1), did not improve the data achieved by the reaction depicted in entry 3 of Table 1.

**Scheme 4:**
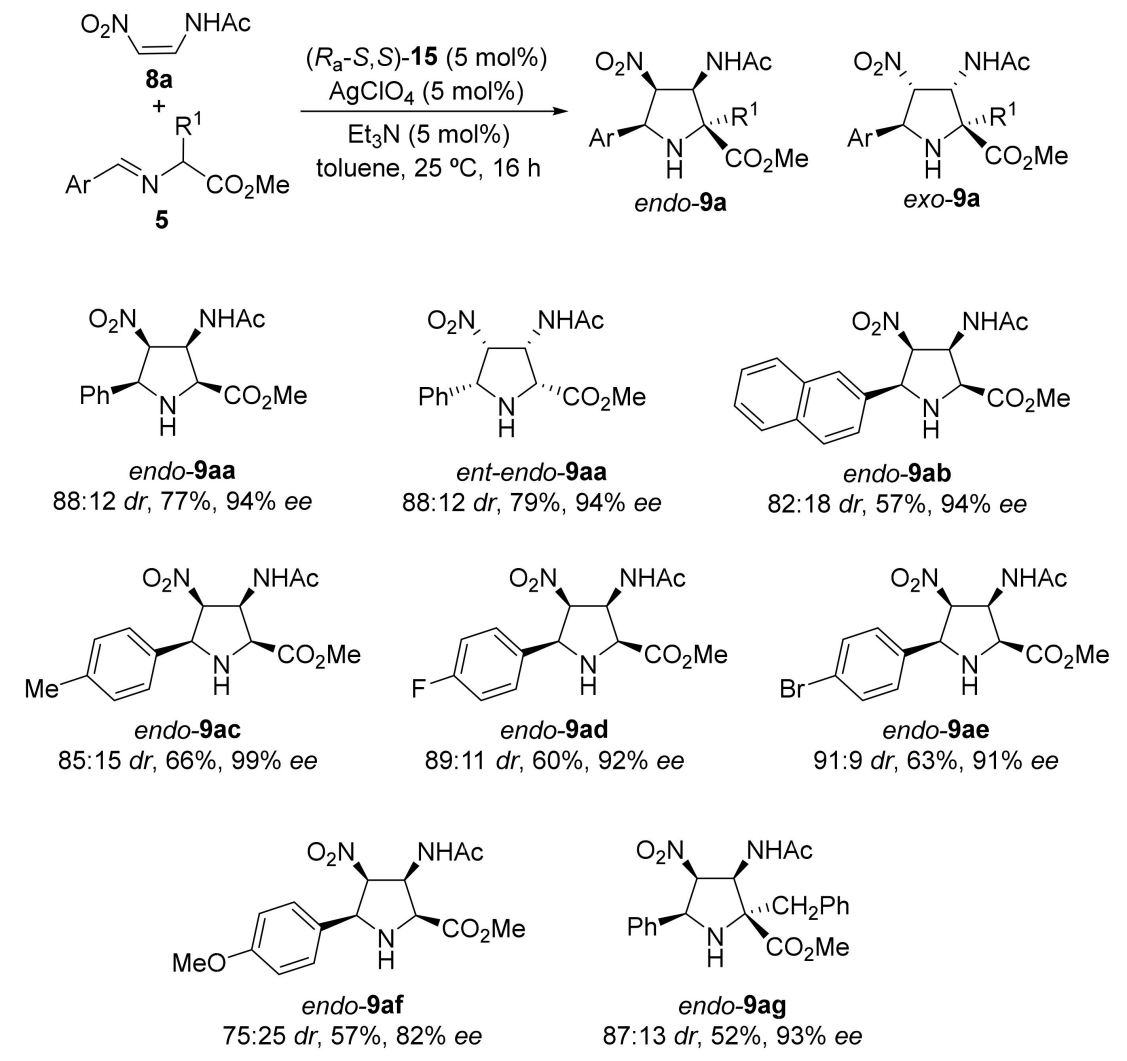
Scope of the enantioselective preparation of *endo*-**9a** under optimized conditions.

As described before, the enantiomerically enriched *ent*-*endo-***9aa** was obtained employing chiral ligand (*S*_a_,*R*,*R*)-**15** in a 79% yield and 94% *ee* (Table 1, entry 3, Scheme 4). The presence of different aromatic substituents at the imino group afforded good yields of the corresponding *endo-*cycloadducts **9aa**–**9af**. In each example, the major diastereoisomer was isolated with a very high enantioselectivity (up to 99% *ee* achieved for *endo*-**9ac**). The phenylalanine-derived imino ester **5g** was also appropriate to run the transformation, generating the prolinate derivative *endo*-**9ag** in a 52% yield, 87:13 *dr*, and with 93% *ee* (Scheme 4).

**Figure 2:**
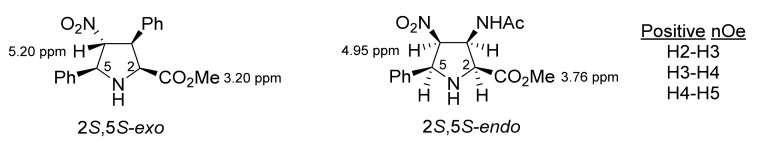
Comparison of ^1^H NMR shifts and nOe data of 2*S*,5*S*-*exo* and 2*S*,5*S*-*endo.*

The absolute configuration of the isolated compounds *endo*-**9a** was assigned according to our previous experience acquired with the synthesis of *exo*-nitroprolinates with these chiral phosphoramidites [45] and based on the nOe experiment data of several examples. Thus, the (2*S*,5*S*)-configuration could be assumed because no appropriate crystals were obtained for X-ray diffraction analysis, and the arrangements of the C3 and C4 were determined by the mentioned results of the nOe tests. Additionally, the characteristic ^1^H NMR chemical shifts of the methyl ester and the H4 atom in the 2*S*,5*S*-*exo* (3.20 and 5.20 ppm, respectively) are completely different to those found in the 2*S*,5*S*-*endo* cycloadduct (3.78–3.85 and 4.95 ppm, respectively) shown in Figure 2.

**Scheme 5:**
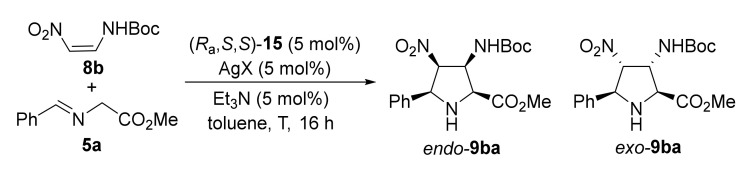
Optimization of the reaction between **5a** and **8b**.

The next cycloaddition was essayed using nitroalkene **8b** because we envisaged a milder hydrolysis and reduction of the carbamate and the nitro groups of *endo-***9b** rather than the hydrolysis of the acetamido unit of cycloadducts *endo*-**9a**. A brief optimization of the selected salts reported in Table 1 was caried out (Scheme 5 and Table 2). Silver perchlorate, under the reaction conditions shown in Scheme 4, afforded a 70:30 *dr* and a 60% *ee* of the *endo*-cycloadduct **9ba** (Table 2, entry 1). Lowering the temperature with this catalytic system was not very fruitful, but the increment in the catalyst loading (10 mol%) produced an increment in both the *dr* and *ee* (73:27 and 68%, respectively) (Table 2, entries 2 and 3). AgOTf, AgTFA, AgOBz, AgSBF_6_, and AgF did not offer noticeable results (Table 2, entries 4, 9–12). However, the silver carbonate·(*R*_a_,*S*,*S*)-**15** combination furnished the highest *ee* (70%) and *dr* (75:25) in a 5 mol% loading and at room temperature, rather than the reaction involving a 2.5 mol% catalyst amount or 0 °C (Table 2, entries 5–7). In the absence of triethylamine, the reaction with silver carbonate was a little bit slow but gave the same result as the obtained one in the reaction with triethylamine (Table 2, entry 8). Again, the copper(I) or copper (II) triflates complexes did not give any conversion (Table 2, entries 13 and 14).

**Scheme 6:**
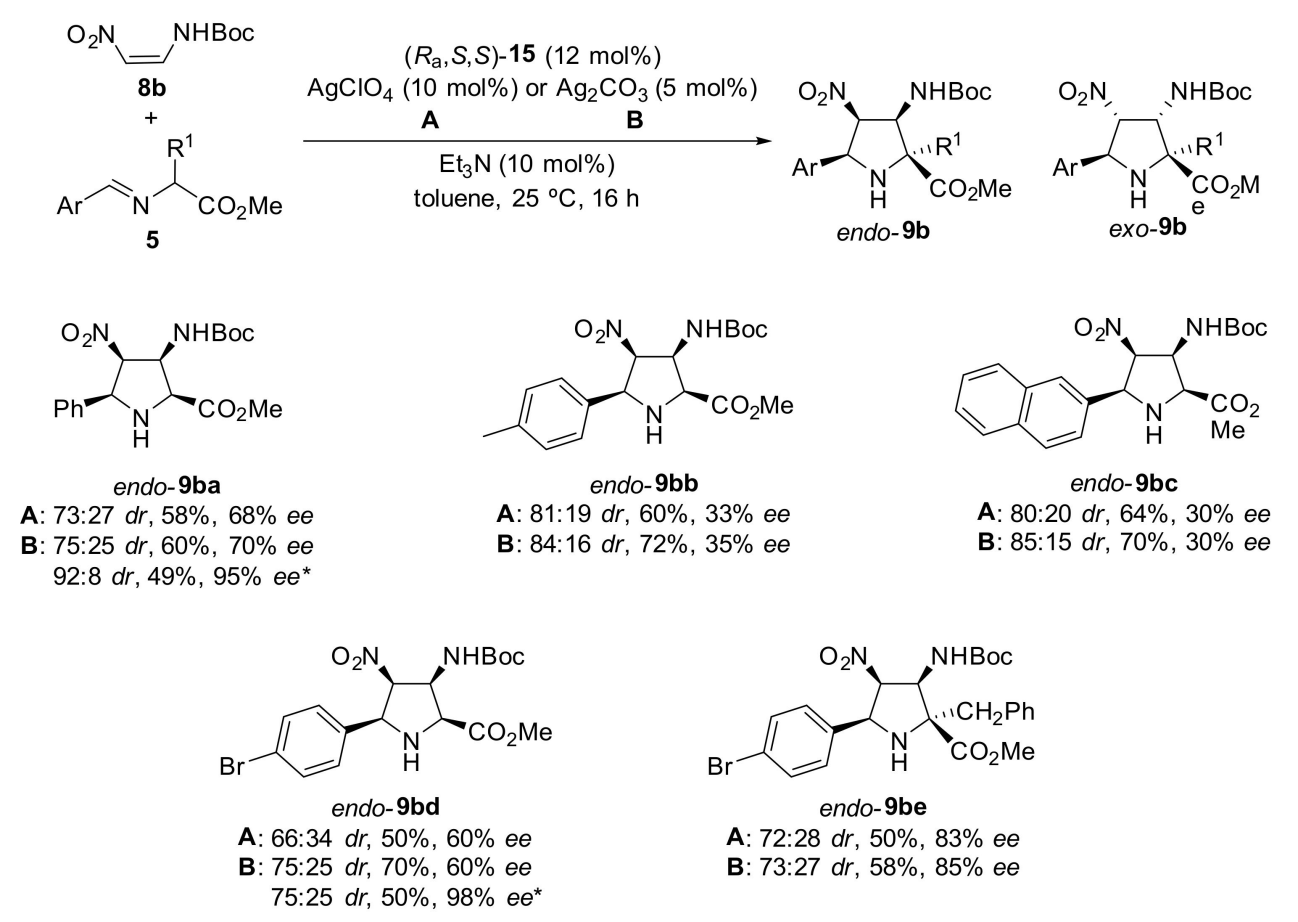
Scope of the enantioselective synthesis of compounds *endo*-**9b** under optimized conditions. * Means enantioselectivity of the mother liquor after separation of the racemic crystals.

The short scope of this 1,3-DC was assessed (Scheme 6). According to the optimization results, two silver salts were tested (Ag_2_CO_3_ and AgClO_4_) for each transformation. In the presence of triethylamine, the reaction was faster. In all of the examples tested, the enantioselectivities and diasteroselectivities were moderated. It was observed that the racemic products precipitated very easily, enriching the resulting mother liquors in the major enantiomer. Thus, very high enantioselections were achieved in compounds *endo-***9ba** and *endo*-**9bd** (Scheme 6). The enantioselectivity obtained in the synthesis of *endo*-**9be** was also very high, without performing a previous crystallization; however, the two diastereoisomers obtained could not be separated by flash chromatography (*exo-*diastereoisomer was the impurity detected in this example).

**Figure 3:**
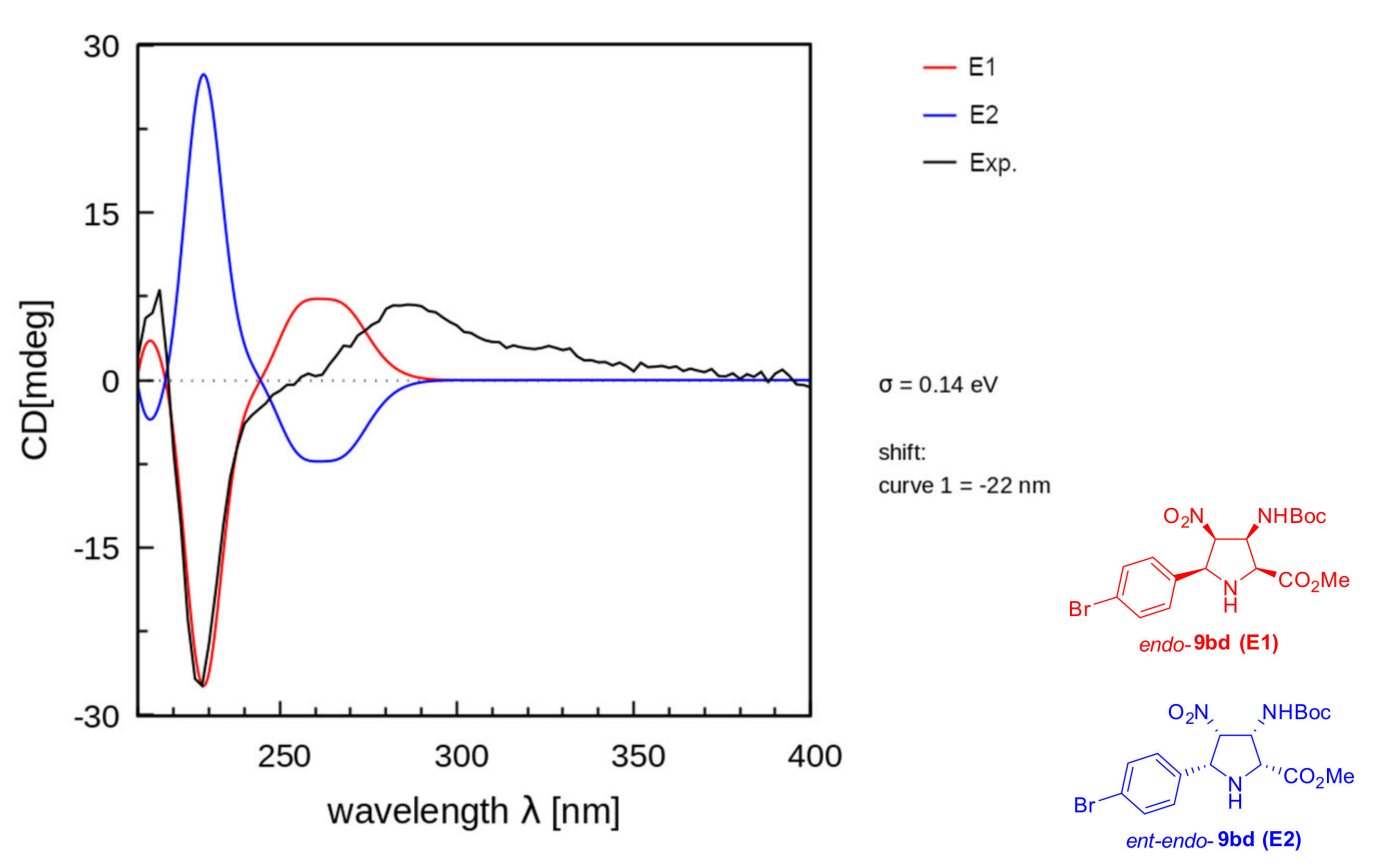
ECD plot comparing both the calculated and experimental spectra for *endo*-**9bd**.

The nOe revealed an identical substituent arrangement for the major stereoisomers depicted in Figure 1 for compounds *all-cis-endo*-**9**. The absolute configuration of *endo*-**9bd** was determined according to electronic circular dichroism (ECD) analysis (Figure 3). The prediction of the ECD spectrum was carried out using the TD-DFT theory through the ORCA 5.0.2 program using the double-hybrid functional B2PLYP and the Def2-TZVP base (see the experimental section). A high correlation was observed between the E1 isomer (red) and the spectrum pilot (black). Although, this correlation is not perfect since a deviation in the dichroism maximum of the band at 270 nm was observed. These deviations are common to the determination of the ECD using the TD-DFT theory. According to the refinement program employed (see the experimental section), the degree of similarity was 67.0% for *endo*-**9bd** (**E1**) and only 0.6% for *ent-endo*-**9bd** (**E2**).

**Scheme 7:**
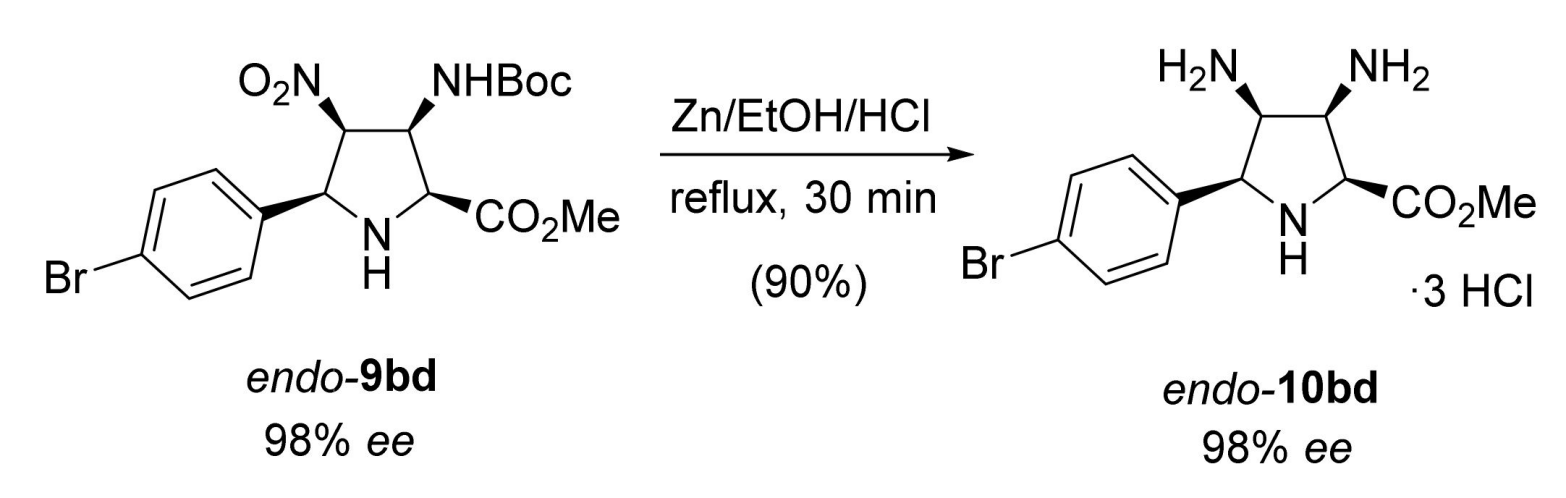
Synthesis of the enantiomerically enriched *cis-*3,4-diamine *endo-***10bd**.

Finally, the synthesis of the target *cis-*3,4-diamine *endo-***10bd** was achieved in only one step involving Zn/concentrated HCl/ethanol under reflux for 30 min (Scheme 7), which did not epimerize enantiomerically enriched similar nitroprolinates [49]. Substrates **9b** were much more suitable for the simultaneous reduction/hydrolysis than the corresponding cycloadducts **9a**. The amido group was very resistant to these acidic conditions.

## 3. Materials and Methods

### 3.1. General

All commercially available reagents and solvents were used without further purification; only aldehydes were distilled prior to use. Analytical TLC was performed on Schleicher & Schuell F1400/LS 254 silica gel plates, and the spots were visualized under UV light (λ = 254 nm). Flash chromatography was carried out on hand-packed columns of Merck silica gel 60 (0.040–0.063 mm). Melting points were determined with a Reichert Thermovar hot plate apparatus and are uncorrected. Optical rotations were measured on a Perkin Elmer 341 polarimeter with a thermally jacketted 5-cm cell at approximately 25 °C and concentrations (*c*) are given in g/100 mL. The structurally most important peaks of the IR spectra (recorded using a Nicolet 510 P-FT) are listed, and wavenumbers are given in cm^−1^. NMR spectra were obtained using a Bruker AC-300 or AC-400 and were recorded at 300 or 400 MHz for ^1^H NMR and 75 or 100 MHz for ^13^C NMR, using CDCl_3_ as the solvent and TMS as the internal standard (0.00 ppm) unless otherwise stated. The following abbreviations are used to describe peak patterns where appropriate: s = singlet, d = doublet, t = triplet q = quartet, m = multiplet or unresolved, and br s = broad signal. All coupling constants (*J*) are given in Hz and chemical shifts in ppm. ^13^C NMR spectra were referenced to CDCl_3_ at 77.16 ppm. In some cases, the small impurities observed in the NMR material correspond with a small proportion of the other diastereoisomer. Low-resolution electron impact (EI) mass spectra were obtained at 70 eV using a Shimadzu QP-5000 by injection or DIP; fragment ions in *m/z* are given with relative intensities (%) in parentheses. High-resolution mass spectra (HRMS) were also carried out in the electron impact mode (EI) at 70 eV using a Finnigan VG Platform or a Finnigan MAT 95S. Enantiomeric excesses were determined using a JASCO-2000 series equipped with a chiral column using mixtures of *n*-hexane/isopropyl alcohol as the mobile phase at 25 °C or with a S_c_CO_2_-HPLC JASCO series 2000. ECD was performed in a Jasco J-810 with a Xe-150 W lamp combined with Gaussian software-DFT calculations (see the Supplementary Materials). Compound **8a**, was prepared according to the published procedure [43,44].

### 3.2. Synthesis of Nitroalkene 8b

To a solution of nitroamine **12** (0.33 M, 440 mg, 5 mmol) in DCM (15 mL), di-*tert-*butyldicarbonate (0.35 M, 1.12 g, 5.5 mmol) was added slowly and the resulting mixture was stirred 24 h at 25 °C. Then, dichloromethane was evaporated, and the residue was purified by flash chromatography, eluting with mixtures of *n-*hexane/ethyl acetate, and affording clean compound **8b** as colorless prisms, mp 81–84 °C (*n*-hexane/EtOAc) (773 mg, 78%). IR (neat) υ_max_: 2980, 1746, 1643, 1338, 732 cm^−1^. ^1^H NMR (300 MHz, CDCl_3_): δ = 1.53 (s, 9H, 3xCH_3_), 6.55 (d, *J* = 6.8 Hz, 1H, CHNO), 7.35 (dd, *J* = 12.6, 6.8 Hz, 1H, C*H*NH), 9.69 (d, *J* = 12.4 Hz, 1H, NH). ^13^C NMR (75 MHz, CDCl_3_): δ = 28.1 (3xCH_3_), 84.2 (*C*Me_3_), 117.2 (CNO), 134.4 (CNH), 151.1 (CO). MS (EI) *m*/*z*: 188 (M^+^, 100%), 189 (8). HRMS (DIP) calculated for C_7_H_12_N_2_O_4_: 188.0797; found 188.0795.

### 3.3. General Experimental Procedure for the Synthesis of α-Imino Esters

The amino ester (1.1 mmol) was dissolved in DCM (2 mL) and the aldehyde (1 mmol) and Et_3_N (1.1 mmol) were added. Then, the mixture was stirred for 16 h at room temperature (25 °C). After, the mixture was quenched by NaCl (saturated aq.), extracted with DCM (3 × 10 mL), and dried with MgSO_4_. The crude residue was obtained after evaporation of the solvent and was used without purification [48].

### 3.4. General Experimental Procedure for the 1,3 Dipolar Cycloaddition of α-Imino Esters and Dipolarophiles

In a flask covered with aluminum foil, the silver salt (see Scheme 4 and Scheme 6), and (*R_a_,S,S*)-**15** (see Scheme 4 and Scheme 6), and toluene (1 mL) were added, and the resulting mixture was stirred for 1 h. Then, a solution of α-imino ester (1 M, 1 mmol) and dipolarophile (1 M, 1 mmol) in toluene (1 mL) was added. To the resulting suspension, triethylamine (0.025 M, 0.05 mmol or 0.05 M, 0.10 mmol) was added and the mixture was stirred at room temperature (25 °C) for 16–24 h. The crude reaction mixture was filtered through a small celite-path and the residue was purified by flash chromatography, yielding pure cycloadducts. The racemic products were formed using 2.5 mol% of (*S_a_,R,R*)-**15** and 2.5 mol% (*R_a_,S,S)*-**15**.

**Methyl (2*S*,3*S*,4*R*,5*S*)-3-acetamido-4-nitro-5-phenylpyrrolidine-2-carboxylate** (*endo*-**9aa**): Purification by flash chromatography (*n*-Hexane-EtOAc 60:40). White foam (236 mg, 77% yield). Enantiomeric excess (94% *ee*) was determined by HPLC. Chiralpak AD-H *n*-hexane/isopropyl alcohol = 90/10, 1.0 mL/min, *t*_Rmin_: 19.0 min, *t*_Rmaj_: 22.5 min, 210.0 nm. IR (neat) υ_max_: 3267, 2954, 1741, 1662, 1550, 1370, 1216, 1032, 730, 699 cm^−1^. ${\left[\alpha \right]}_{D}^{30}$ = 8.7 (*c* 1.2, CHCl_3_); ^1^H NMR (300 MHz, CDCl_3_): δ = 1.95 (s, 3H, C*H*_3_), 3.01 (br s, 1H, N*H*), 3.76 (s, 3H, OC*H*_3_), 4.44 (d, *J* = 7.1 Hz, 1H, NHC*H*COOMe), 4.72 (d, *J* = 7.2 Hz, 1H, NHC*H*Ph), 4.95 (dd, *J* = 7.2, 5.3 Hz, 1H, NO_2_C*H*), 5.26 (ddd, *J* = 8.5, 7.2, 5.3 Hz, 1H, NHC*H*CHNO_2_), 6.62 (d, *J* = 8.5 Hz, 1H, N*H*COCH_3_), 7.28–7.62 (m, 5H, Ar*H*). ^13^C NMR (101 MHz, CDCl_3_): δ = 22.9 (*C*H_3_), 53.0 (NH*C*HCO), 55.7 (O*C*H_3_), 61.9 (*C*NHCO), 65.2 (Ph*C*HNH), 93.8 (*C*NO_2_), 127.3, 127.4, 129.3, 129.5 (Ar*C*), 169.2, 171.0 (2x*C*=O). MS (EI) *m*/*z*: 259 (M^+^-NO_2_, 5%), 202 (41), 201 (100), 177 (23), 174 (93), 170 (17), 160 (15), 159 (45), 158 (12), 143 (23), 142 (40), 132 (85), 117 (68), 155 (49), 103 (14), 91 (12), 77 (17), 43 (35). HRMS (DIP) calculated for C_14_H_17_N_3_O_5_: 307.1168; found: 307.1173.

**Methyl (2*R*,3*R*,4*S*,5*R*)-3-acetamido-4-nitro-5-phenylpyrrolidine-2-carboxylate** (*ent-endo*-**9aa**): Purification by flash chromatography (*n*-Hexane-EtOAc 6:4). White foam (242 mg, 79% yield). Enantiomeric excess (94% *ee*) was determined by HPLC. Chiralpak AD-H *n*-hexane/isopropyl alcohol = 90/10, 1.0 mL/min, *t*_Rmaj_: 19.0 min, *t*_Rmin_: 22.5 min, 210.0 nm. ${\left[\alpha \right]}_{D}^{30}$ = −8.8 (*c* 1.2, CHCl_3_).

**Methyl (2*S*,3*S*,4*R*,5*S*)-3-acetamido-5-(naphth-2-yl)-4-nitropyrrolidine-2-carboxylate** (*endo*-**9ab**): Purification by flash chromatography (*n*-Hexane-EtOAc 5:5). Pale yellow foam (203 mg, 57% yield). Enantiomeric excess (91% *ee*) was determined by HPLC. Chiralpak AD-H *n*-hexane/isopropyl alcohol = 90/10, 1.0 mL/min, *t*_Rmin_: 31.5 min, *t*_Rmaj_: 43.4 min, 220.0 nm. IR (neat) υ_max_: 3271, 1741, 1662, 1550, 1369, 860 cm^−1^. ${\left[\alpha \right]}_{D}^{32}$ = 32.1 (*c* 0.75, CHCl_3_). ^1^H NMR (300 MHz, CDCl_3_): δ = 1.95 (s, 3H, C*H*_3_), 3.71 (s, 3H, OC*H*_3_), 4.53 (d, *J* = 7.2 Hz, 1H, NHC*H*COOMe), 4.92 (d, *J* = 7.3 Hz, 1H, NHC*H*Ph), 5.08 (dd, *J* = 7.3, 5.1 Hz, 1H, NO_2_C*H*), 5.33 (ddd, *J* = 8.5, 7.2, 5.0 Hz, 1H, NHC*H*CHNO_2_), 6.72 (d, *J* = 8.8 Hz, 1H, N*H*COCH_3_), 7.47–7.54 (m, 2H, Ar*H*), 7.57 (dd, *J* = 8.5, 1.8 Hz, Ar*H*), 7.80–7.94 (m, 4H, Ar*H*). ^13^C NMR (101 MHz, CDCl_3_): δ =23.0 (*C*H_3_), 52.8 (NH*C*HCO), 55.9 (O*C*H_3_), 61.8 (*C*NHCO), 65.7 (Ph*C*HNH), 94.3 (*C*NO_2_), 124.1, 126.6, 126.8, 127.9, 128.2, 129.3, 133.2, 133.6 (Ar*C*), 170.3, 170.5 (2x*C*=O). MS (EI) *m*/*z*: 311 (M^+^-NO_2_, 5%), 252 (51), 251 (83), 227 (50), 224 (33), 220 (25), 219 (18), 209 (25), 208 (11), 196 (21), 193 (24), 182 (30), 180 (44), 168 (25), 167 (82), 166 (29), 165 (100), 153 (14), 152 (26), 140 (14), 43 (32). HRMS (DIP) calculated for C_18_H_19_N_3_O_5_: 357.1325; found: 357.1318.

**Methyl (2*S*,3*S*,4*R*,5*S*)-3-acetamido-4-nitro-5-(*p*-tolyl)pyrrolidine-2-carboxylate** (*endo*-**9ac**): Purification by flash chromatography (*n*-Hexane-EtOAc 5:5). White foam (212 mg, 66% yield). Enantiomeric excess (99% *ee*) was determined by HPLC. Chiralpak IA *n*-hexane/isopropyl alcohol = 90/10, 1.0 mL/min, *t*_Rmin_: 51.1 min, *t*_Rmaj_: 57.3 min, 220.0 nm. IR (neat) υ_max_: 3264, 1742, 1661,1369, 1214, 818, 735 cm^−1^.$\;{\left[\alpha \right]}_{D}^{30}$ = 8.3 (*c* 1.0, CHCl_3_); ^1^H NMR (300 MHz, CDCl_3_): δ = 1.95 (s, 3H, C*H*_3_CO), 2.34 (s, 3H, C*H*_3_), 2.96 (br s, 1H, N*H*), 3.76 (s, 3H, OC*H*_3_), 4.43 (d, *J* = 7.7 Hz, 1H, NHC*H*COOMe), 4.68 (d, *J* = 7.6 Hz, 1H, NHC*H*Ph), 4.94 (dd, *J* = 7.2, 5.3 Hz, 1H, NO_2_C*H*), 5.26 (ddd, *J* = 8.5, 7.2, 5.2 Hz, 1H, NHC*H*CHNO_2_), 6.65 (d, *J* = 8.5 Hz, 1H, N*H*COCH_3_), 7.18 (d, *J* = 8.0 Hz, 2H, Ar*H*), 7.33 (d, *J* = 8.0 Hz, 2H, Ar*H*). ^13^C NMR (101 MHz, CDCl_3_): δ =21.3 (*C*H_3_), 22.9 (*C*H_3_), 53.0 (NH*C*HCO), 55.7 (O*C*H_3_), 61.9 (*C*NHCO), 65.2 (Ph*C*HNH), 93.8 (*C*NO_2_), 127.3, 130.0, 139.6 (Ar*C*), 169.1, 171.1 (2x*C*=O). MS (EI) *m*/*z*: 275 (M^+^-NO_2_, 4%), 217 (10), 216 (53), 215 (100), 191 (32), 189 (13), 188 (84), 184 (24), 183 (18), 174 (12), 173 (35), 172 (11), 159 (12), 157 (27), 156 (64), 149 (17), 146 (64), 144 (26), 132 (14), 131 (82), 130 (36), 129 (58), 128 (12), 115 (22), 91 (20), 57 (14), 43 (53). HRMS (DIP) calculated for C_15_H_19_N_3_O_5_: 321.1325; found: 321.1326.

**Methyl (2*S*,3*S*,4*R*,5*S*)-3-acetamido-5-(4-fluorophenyl)-4-nitropyrrolidine-2-carboxylate** (*endo*-**9ad**): Purification by flash chromatography (*n*-Hexane-EtOAc 6:4). White foam (195 mg, 60% yield). Enantiomeric excess (92% *ee*) was determined by HPLC. Chiralpak AD-H *n*-hexane/isopropyl alcohol = 90/10, 1.0 mL/min, *t*_Rmin_: 19.5 min, *t*_Rmaj_: 25.8 min, 220.5 nm. IR (neat) υ_max_: 3257, 1743, 1553, 1225, 838, 729 cm^−1^.$\;{\left[\alpha \right]}_{D}^{30}$ = 6.7 (*c* 0.95, CHCl_3_); ^1^H NMR (300 MHz, CDCl_3_): δ = 1.96 (s, 3H, C*H*_3_), 2.96 (br s, 1H, N*H*), 3.77 (s, 3H, OC*H*_3_), 4.43 (d, *J* = 7.3 Hz, 1H, NHC*H*COOMe), 4.71 (d, *J* = 7.4 Hz, 1H, NHC*H*Ph), 4.93 (dd, *J* = 7.4, 5.7 Hz, 1H, NO_2_C*H*), 5.26 (ddd, *J* = 8.4, 7.3, 5.7 Hz, 1H, NHC*H*CHNO_2_), 6.65 (d, *J* = 8.4 Hz, 1H, N*H*COCH_3_), 7.06 (m, 2H, Ar*H*), 7.45 (m, 2H, Ar*H*). ^19^F NMR (300 MHz, CDCl_3_): δ =−115.37 ppm. ^13^C NMR (101 MHz, CDCl_3_): (*C*H_3_), 53.3 (NH*C*HCO), 55.1 (O*C*H_3_), 61.9 (*C*NHCO), 64.0 (Ph*C*HNH), 92.8 (*C*NO_2_), 116.3, 116.6, 130.1, 130.2, 162.4, 164.9 (Ar*C*), 167.8, 171.8 (2x*C*=O). MS (EI) *m*/*z*: 279 (M^+^-NO_2_, 4%), 277 (13), 220 (38), 219 (98), 195 (24), 192 (90), 188 (16), 187 (13), 178 (15), 177 (48), 176 (14), 175 (12), 161 (26), 160 (55), 150 (100), 148 (31), 135 (75), 133 (37), 108 (16), 101 (15), 43 (60). HRMS (DIP) calculated for C_14_H_16_FN_3_O_5_: 325.1074; found: 325.1076.

**Methyl (2*S*,3*S*,4*R*,5*S*)-3-acetamido-5-(4-bromophenyl)-4-nitropyrrolidine-2-carboxylate** (*endo*-**9ae**): Purification by flash chromatography (*n*-Hexane-EtOAc 6:4). White foam (180 mg, 63% yield). Enantiomeric excess (91% *ee*) was determined by HPLC. Chiralpak AD-H *n*-hexane/isopropyl alcohol = 90/10, 1.0 mL/min, *t*_Rmin_: 21.4 min, *t*_Rmaj_: 26.8 min, 220.5 nm. IR (neat) υ_max_: 3259, 1740, 1662, 1549, 1369, 1215, 1010, 819 cm^−1^.$\;{\left[\alpha \right]}_{D}^{28}$ = 5.21 (*c* 1.0, CHCl_3_); ^1^H NMR (300 MHz, CDCl_3_): δ = 1.96 (s, 3H, C*H*_3_), 2.98 (br s, 1H, N*H*), 3.77 (s, 3H, OC*H*_3_), 4.47 (d, *J* = 7.2 Hz, 1H, NHC*H*COOMe), 4.74 (d, *J* = 7.3 Hz, 1H, NHC*H*Ph), 4.94 (dd, *J* = 7.3, 5.5 Hz, 1H, NO_2_C*H*), 5.19–5.33 (m, 1H, NHC*H*CHNO_2_), 6.63 (d, *J* = 8.5 Hz, 1H, N*H*COCH_3_), 7.36 (d, *J* = 8.5 Hz, 2H, Ar*H*), 7.51 (d, *J* = 8.5 Hz, 2H, Ar*H*). ^13^C NMR (101 MHz, CDC_3_): δ =22.9 (*C*H_3_), 53.3 (NH*C*HCO), 55.1 (O*C*H_3_), 61.9 (*C*NHCO), 64.0 (Ph*C*HNH), 92.7 (*C*NO_2_), 124.2, 129.7, 132.5 (Ar*C*), 168.0, 171.6 (2x*C*=O). MS (EI) *m*/*z*: 339 (M^+^-NO_2_, 7%), 282 (48), 281 (99), 280 (47), 179 (100), 257 (27), 255 (41), 254 (90), 252 (96), 250 (13), 248 (13), 239 (36), 237 (40), 222 (37), 220 (37), 212 (70), 210 (82), 197 (41), 195 (59), 130 (36), 116 (29), 43 (90). HRMS (DIP) calculated for C_12_H_13_BrN_2_O [M-C_2_H_3_NO_3_]: 279.9946; found: 279.9933.

**Methyl (2*S*,3*S*,4*R*,5*S*)-3-acetamido-5-(4-methoxyphenyl)-4-nitropyrrolidine-2-carboxylate** (*endo*-**9af**): Purification by flash chromatography (*n*-Hexane-EtOAc 5:5). Pale yellow liquid (183 mg, 57% yield). Enantiomeric excess (82% *ee*) was determined by HPLC. Chiralpak AD-H *n*-hexane/isopropyl alcohol = 90/10, 1.0 mL/min, *t*_Rmin_: 28.5 min, *t*_Rmaj_: 34.4 min, 220.0 nm. IR (neat) υ_max_: 3272, 1740, 1662, 1551, 1248, 833, 765 cm^−1^. ${\left[\alpha \right]}_{D}^{29}$ = 10.46 (*c* 1.3, CHCl_3_); ^1^H NMR (300 MHz, CDCl_3_): δ = 1.97 (s, 3H, C*H*_3_CO), 3.76 (s, 3H, OC*H*_3_), 3.80 (s, 3H, OC*H*_3_),4.47 (d, *J* = 7.3 Hz, 1H, NHC*H*COOMe), 4.70 (d, *J* = 7.5 Hz, 1H, NHC*H*Ph), 4.96 (dd, *J* = 7.5, 5.2 Hz, 1H, NO_2_C*H*), 5.20–5.30 (m, 1H, NHC*H*CHNO_2_), 6.72 (d, *J* = 8.6 Hz, 1H, N*H*COCH_3_), 6.90 (d, *J* = 8.8 Hz, 2H, Ar*H*), 7.39 (d, *J* = 8.7 Hz, 2H, Ar*H*). ^13^C NMR (75 MHz, CDCl_3_): δ =23.0 (*C*H_3_), 52.9 (NH*C*HCO), 55.5 (O*C*H_3_), 55.9 (O*C*H_3_), 61.8 (*C*NHCO), 65.3 (Ph*C*HNH), 94.3 (*C*NO_2_), 114.6, 128.4, 160.3 (Ar*C*), 170.2, 170.6 (2x*C*=O). MS (EI) *m*/*z*: 292 (M^+^-NO_2_, 7%), 291 (39), 289 (39), 288 (14), 258 (13), 257 (15), 233 (17), 232 (100), 231 (42), 216 (16), 215 (12), 207 (13), 204 (19), 200 (18), 199 (14), 185 (23), 180 (18), 173 (14), 172 (19), 162 (47), 160 (17), 147 (20), 146 (19), 145 (47), 143 (47), 135 (20), 121 (28), 43 (32). HRMS (DIP) calculated for C_15_H_19_N_3_O_5_: 321.1325; found: 321.1326.

**Methyl (2*S*,3*S*,4*R*,5*S*)-3-acetamido-2-benzyl-4-nitro-5-phenylpyrrolidine-2-carboxylate** (*endo*-**9ag**): Purification by flash chromatography (*n*-Hexane-EtOAc 6:4). Pale yellow sticky foam (52% yield). Enantiomeric excess (93% *ee*) was determined by HPLC. Chiralpak AD-H *n*-hexane/isopropyl alcohol = 90/10, 1.0 mL/min, *t*_Rmaj_: 12.6 min, *t*_Rmin_: 20.3 min, 220.0 nm. IR (neat) υ_max_: 3267, 1746, 1667, 1559, 1237, 833, 731 cm^−1^.$\;{\left[\alpha \right]}_{D}^{28}$ = 12.43 (*c* 1.2, CHCl_3_); ^1^H NMR (300 MHz, CDCl_3_): δ = 1.90 (s, 3H, C*H*_3_CO), 3.29 (s, 2H, C*H*_2_), 3.78 (s, 3H, OC*H*_3_), 4.65 (d, *J* = 9.9 Hz, 1H, NHC*H*Ph), 4.91–5.07 (m, 1H, NO_2_C*H*), 5.38 (t, *J* = 9.4 Hz, 1H, NHC*H*CHNO_2_), 6.71 (d, *J* = 9.7 Hz, 1H, N*H*COCH_3_), 7.26–7.35 (m, 10H, Ar*H*). ^13^C NMR (75 MHz, CDCl_3_): δ = 23.2 (*C*H_3_), 41.4 (NH*C*CO), 52.9 (O*C*H_3_), 59.9 (*C*NHCO), 63.1 (Ph*C*HNH), 93.8 (*C*NO_2_), 126.7, 127.7, 128.8, 128.9, 129.1, 129.3, 130.5, 134.3, 138.2 (Ar*C*), 170.3, 173.7 (2x*C*=O). MS (EI) *m*/*z*: 397 (M^+^, 1%), 306 (22), 291 (20), 259 (47), 232 (25), 228 (15), 227 (95), 217 (23), 186 (13), 185 (100), 174 (18), 158 (17), 157 (100), 132 (23), 130 (19), 115 (19), 91 (53), 43 (19). HRMS (DIP) calculated for C_21_H_23_N_3_O_5_: 397.4218; found: 397.4212.

**Methyl (2*S*,3*S*,4*R*,5*S*)-3-[(*tert*-butoxycarbonyl)amino]-4-nitro-5-phenylpyrrolidine-2-carboxylate** (*endo*-**9ba**): Purification by flash chromatography (*n*-Hexane-EtOAc 4:1). Pale yellow foam, mp: 82–85 °C (*n*-Hexane-EtOAc), (178 mg, 49% yield). Enantiomeric excess (95% *ee*) was determined by HPLC. Chiralpak IA *n*-hexane/isopropyl alcohol = 90/10, 1.0 mL/min, *t*_Rmin_: 21.8 min, *t*_Rmaj_: 31.5 min, 210.0 nm. IR (neat) υ_max_: 3359, 3289, 2985, 1739, 1685, 1546, 1365, 1168, 744 cm^−1^.$\;{\left[\alpha \right]}_{D}^{28}$ = -14.63 (*c* 1.0, CHCl_3_); ^1^H NMR (300 MHz, CDCl_3_): δ = 1.42 (s, 9H, 3xCH_3_), 2.60 (br s, 1H, NH), 3.79 (s, 3H, OMe), 4.45–4.32 (m, 1H, CHCO), 4.75–4.60, 4.93–4.78 (m, 2H, 2xCHN), 5.03 (dd, *J* = 14.4, 6.2 Hz, 1H, CHNO), 5.25–5.11 (m, 1H, NHCO), 7.48–7.30 (m, 5H, Ar*H*). ^13^C NMR (75 MHz, CDCl_3_): δ = 28.3 (3xCH_3_), 52.7 (OMe), 57.7, 62.1, 65.9 (3xCHN), 80.9 (*C*Me_3_), 95.1 (CHNO), 126.8, 128.9, 129.2, 138.4 (Ar*C*), 154.79, 171.08 (2xCO). MS (EI) *m/z*: 365 (M^+^, 10%), 236 (18), 177 (16), 91 (12), 77 (100). HRMS (DIP) calculated for C_17_H_23_N_3_O_6_: 365.1587; found: 365.1582.

**Methyl (2*S*,3*S*,4*R*,5*S*)-3-[(*tert*-butoxycarbonyl)amino]-4-nitro-5-(*p*-tolyl)pyrrolidine-2-carboxylate** (*endo*-**9bb**): Purification by flash chromatography (*n*-Hexane-EtOAc 4:1). Pale yellow foam, mp: 72–75 °C (*n*-hexane-EtOAc); (273 mg, 72% yield). Enantiomeric excess (35% *ee*) was determined by S_c_CO_2_-HPLC. Chiralpak IC CO_2_/ethyl alcohol = 2.8/0.2, 3.0 mL/min, *t*_Rmin_: 6.2 min, *t*_Rmaj_: 6.8 min, 210 nm. IR (neat) υ_max_: 3336, 2977, 2927, 1709, 1550, 1515, 1446, 1365, 1164, 813, 775 cm^−1^.$\;{\left[\alpha \right]}_{D}^{28}$ = -5.9 (*c* 0.9, CHCl_3_). ^1^H NMR (300 MHz, CDCl_3_): δ = 1.44 (s, 9H, 3xCH_3_), 2.36 (s, 3H, Ar*Me*), 2.65 (br s, 1H, NH), 3.81 (s, 3H, OMe), 4.38 (d, *J* = 7.0 Hz, 1H, CHCO), 4.63 (d, *J* = 7.2 Hz, 1H, C*H*Ar), 4.94–4.81, 5.11–4.99 (2m, 2H, CHNCO and CHNO), 5.17 (d, *J* = 9.9 Hz, 1H, NHCO), 7.23–7.12 (m, 2H, Ar*H*), 7.32 (d, *J* = 8.0 Hz, 2H, Ar*H*). ^13^C NMR (101 MHz, CDCl_3_): δ = 21.3 (ArC*H*_3_), 28.3 (3xCH_3_), 52.6 (OMe), 57.8, 62.2, 65.8 (3xCHN), 80.9 (*C*Me_3_), 95.3 (CHNO), 126.7, 129.8, 135.3, 138.8 (Ar*C*), 154.8, 171.1 (2xCO). MS (EI) *m*/*z*: 379 (M^+^, 5%), 279 (48), 218 (24), 189 (86), 91 (100). HRMS (DIP) calculated for C_18_H_25_N_3_O_6_: 379.0750; found: 379.0742.

**Methyl (2*S*,3*S*,4*R*,5*S*)-3-[(*tert*-butoxycarbonyl)amino]-4-nitro-5-(2-naphthyl)pyrrolidine-2-carboxylate (***endo***-9bc):** Purification by flash chromatography (*n*-Hexane-EtOAc 4:1). Pale yellow prisms, mp: 79–83 °C (*n*-Hexane-EtOAc); (290 mg, 70% yield). Enantiomeric excess (30% *ee*) was determined by S_c_CO_2_-HPLC. Chiralpak IA CO_2_/ethyl alcohol = 2.7/0.3, 3.0 mL/min, *t*_Rmin_: 12.3 min, *t*_Rmaj_: 19.8 min, 226.7 nm. IR (neat) υ_max_: 3351, 2973, 2921, 2337, 1712, 1667, 1550, 1500, 1365, 1253, 1161, 821, 736 cm^−1^.$\;{\left[\alpha \right]}_{D}^{28}$ = 1.53 (*c* 0.9, CHCl_3_). ^1^H NMR (300 MHz, CDCl_3_): δ = 1.42 (s, 9H, 3xCH_3_), 2.75 (br s, 1H, NH), 3.81 (s, 3H, OMe), 4.43 (d, *J* = 7.0 Hz, 1H, CHCO), 4.83 (d, *J* = 6.9 Hz, 1H, C*H*Ar), 4.99–4.90 (m, 1H, CHNCO), 5.06 (dt, *J* = 13.2, 6.8 Hz, 1H, CHNO), 5.30–5.17 (m, 1H, NHCO), 7.62–7.42 (m, 3H, Ar*C*), 7.97–7.75 (m, 4H, Ar*C*). ^13^C NMR (75 MHz, CDCl_3_): δ = 28.3 (3xCH_3_), 52.7 (OMe), 57.8, 62.2, 65.9 (3xCHN), 80.8 (*C*Me_3_), 95.1 (CHNO), 124.1, 126.2, 126.7, 126.7, 127.8, 128.2, 129.2, 133.3, 133.6 (Ar*C*), 151.5, 171.1 (2xCO). MS (EI) *m/z*: 415 (M^+^, 2%), 370 (22), 314 (18), 281 (12), 264 (43), 251 (100), 207 (33), 190 (56), 131 (34), 91 (90). HRMS (DIP) calculated for C_21_H_25_N_3_O_6_: 415.1720; found: 415.1712.

**Methyl (2*S*,3*S*,4*R*,5*S*)-5-(4-bromophenyl)-3-[(*tert*-butoxycarbonyl)amino]-4-nitropyrrolidine-2-carboxylate** (*endo*-**9bd**): Purification by flash chromatography (*n*-Hexane-EtOAc 4:1). Colourless needles, mp: 72–75 °C (*n*-Hexane-EtOAc); (222 mg, 50% yield). Enantiomeric excess (98% *ee*) was determined by S_c_CO_2_-HPLC. Chiralpak IC CO_2_/ethyl alcohol = 2.8/0.2, 3.0 mL/min, *t*_Rmin_: 7.8 min, *t*_Rmaj_: 10.2 min, 210 nm. IR (neat) υ_max_: 3336, 2978, 1708, 1550, 1515, 1365, 1237, 1165, 823, 736 cm^−1^.$\;{\left[\alpha \right]}_{D}^{28}$ = −4.5 (*c* 1.0, CHCl_3_). ^1^H NMR (300 MHz, CDCl_3_): δ = 1.41 (s, 9H, 3xCH_3_), 2.25 (br s, 1H, NH), 3.80 (s, 3H, OMe), 4.37 (d, *J* = 7.1 Hz, 1H, CHCO), 4.65 (d, *J* = 7.1 Hz, 1H, C*H*Ar), 4.89–4.76 (m, 1H, CHNCO), 5.07–4.94 (m, 1H, CHNO), 5.19–5.09 (m, 1H, NHCO), 7.33 (d, *J* = 8.4 Hz, 2H, Ar*H*), 7.57–7.45 (m, 2H, Ar*H*). ^13^C NMR (126 MHz, CDCl_3_): δ = 28.3 (3xCH_3_), 52.7 (OMe), 57.7, 61.9, 65.0 (3xCHN), 81.1 (*C*Me_3_), 94.9 (CNO), 122.9, 128.6, 132.3, 137.7 (Ar*C*), 154.8, 171.1 (2xCO). MS (EI) *m*/*z*: 445, 443 (M^+^, 0.12%), 370 (22), 314 (18), 282 (20), 281 (53), 257 (100), 255 (100), 225 (30), 223 (30), 212 (87), 131 (34), 91 (50). HRMS (DIP) calculated for C_17_H_22_BrN_3_O_6_: 444.0692; found: 444.0682.

**Methyl (2*S*,3*R*,4*R*,5*S*)-2-benzyl-5-(4-bromophenyl)-3-[(*tert*-butoxycarbonyl)amino]-4-nitropyrrolidine-2-carboxylate** (*endo*-**9be**)**:** Purification by flash chromatography (*n*-Hexane-EtOAc 4:1, impurified with the *exo*-diastereoisomer). Colourless prisms, mp: 73–76 °C (*n*-Hexane-EtOAc); (309 mg, 58% yield). Enantiomeric excess (85% *ee*) was determined by S_c_CO_2_-HPLC. Chiralpak IA CO_2_/ethyl alcohol = 2.8/0.2, 3.0 mL/min, *t*_Rmaj_: 10.3 min, *t*_Rmin_: 16.8 min, 210 nm. IR (neat) υ_max_: 3352, 2978, 1712, 1550, 1496, 1365, 1257, 1160, 825, 736 cm^−1^.$\;{\left[\alpha \right]}_{D}^{28}$ = 17.4 (*c* 0.8, CHCl_3_). ^1^H NMR (400 MHz, CDCl_3_): δ = 1.45 (s, 9H, 3xCH_3_), 2.55 (br s, 1H, NH), 3.23 (m, 1H, C*H*_2_Ph), 3.33 (d, *J* = 15.0 Hz, 1H, C*H*_2_Ph), 3.79 (s, 3H, OMe), 4.46 (d, *J* = 8.4 Hz, 1H, CHCO), 4.65 (d, *J* = 7.1 Hz, 1H, C*H*Ar), 4.85 (t, *J* = 8.8 Hz, 1H, CHNCO), 4.98–5.14 (m, 1H, CHNO), 5.31 (d, *J* = 9.9 Hz, 1H, NHCO), 7.17 (dd, *J* = 15.5, 7.4 Hz, 2H, Ar*H*), 7.38–7.27 (m, 5H, Ar*H*), 7.49–7.40 (m, 2H, Ar*H*). ^13^C NMR (126 MHz, CDCl_3_): δ = 28.2 (3xCH_3_), 40.6 (CH_2_), 52.8 (OMe), 61.5, 62.3, 69.7 (2xCHN and CBn), 80.7 (*C*Me_3_), 94.1 (CNO), 122.6, 127.5, 128.2, 128.6, 129.7, 130.5, 134.3, 137.8 (Ar*C*), 154.7, 173.5 (2xCO). MS (EI) *m/z*: 535, 533 (M^+^, 0.02%), 454 (67), 370 (22), 314 (18), 131 (34), 91 (100). HRMS (DIP) calculated for C_24_H_29_N_3_O_6_: 534.2056; found: 534.2055.

### 3.5. General Procedure for the Synthesis of Methyl (2S,3R,4S,5S)-3,4-Diamino-2-benzyl-5-(4-bromophenyl)pyrrolidine-2-carboxylate trihydrochloride (endo-10be)

To a flask containing *endo*-**9be** (266 mg, 0.5 mmol) and zinc powder (163 mg, 2.5 mmol) in absolute ethanol (5 mL), concentrated hydrochloric acid (5 mL) was slowly added. The resulting suspension was stirred and refluxed for 30 min. Then, the mixture was filtered through a celite path, and the solution was evaporated to dryness. The pale-yellow oil was washed with diethyl ether (2x5 mL), affording 230 mg (90% yield) of *endo*-**10be**. IR (neat) υ_max_: 3322, 2973, 2923, 1705, 1550, 1519, 1446, 1365, 1165, 814 cm^−1^.$\;{\left[\alpha \right]}_{D}^{28}$ = 20.55 (*c* 0.5, MeOH). ^1^H NMR (300 MHz, methanol-*d*_4_): δ = 3.81 (s, 3H, OMe), 4.03 (dd, *J* = 8.5, 4.7 Hz, 1H, C*H*NH_2_) 4.45 (dd, *J* = 7.3, 4.7 Hz, 1H, C*H*NH_2_), 4.51 (d, *J* = 8.5 Hz, 1H, CHCO), 4.70 (d, *J* = 7.3 Hz, 1H, C*H*Ar), 7.55 (m, 4H, Ar*H*). ^13^C NMR (75 MHz, methanol-*d*_4_): δ = 53.9 (OMe), 54.9, 60.6, 60.7, 65.0 (4xCHN), 124.7, 131.6, 133.3, 135.1 (Ar*C*), 168.7 (CO). MS (EI) *m*/*z*: 315, 313 (M^+^, 0.02%), 284 (15), 283 (50), 282 (83), 281 (96), 280 (70), 279 (42), 251 (51), 250 (88), 249 (100), 248 (84), 247 (59), 224 (30), 223 (40), 222 (42), 221 (42), 207 (83), 193 (32). HRMS (DIP) calculated for C_12_H_16_BrN_3_O_2_: 314.0416; found: 314.0410.

## 4. Conclusions

In this work, a high enantioslective synthesis of nitroprolinates containing a vicinal potential amino group was reported. The best combination of the catalyst precursor was the chiral phosphoramidite together with silver perchlorate with the (*Z*)-nitroacetamide or with silver perchlorate/silver carbonate for the (*Z*)-nitrocarbamate. The *all-cis* arrangement of all functional groups attached to the pyrrolidine ring and their absolute configuration were determined by ECD. This methodology was appropriate to obtain enantiomerically enriched 1,2-*cis*-diamines, which can be employed in many scientific areas. For this purpose, the carbamate was hydrolyzed using milder reaction conditions than the corresponding pyrrolidines bearing the acetamido group.

## Figures and Tables

**Table 1:** Results of the optimization of the 1,3-DC of **5a** and **8a**.

Entry	Metal Salt	Ligand	*endo:exo* ^1^	Yield (%) ^2^	*Ee* ^3^
1	AgClO_4_	(*S*_a_)-**13**	--	<10	<−35
2	AgClO_4_	(*S*_a_)-**14**	--	<15	<−30
3	AgClO_4_	(*S*_a_,*R*,*R*)-**15**	88:12	77	−94
4	AgClO_4_	(*S*_a_)-**16a**	--	<10	--
5	AgClO_4_	(*S*_a_)-**16b**	--	<10	--
6	AgClO_4_	(*S*_a_)-**16c**	--	<10	--
7 ^4^	Cu(OTf)	(*S*_a_)-**16a**	--	<5	--
8 ^4^	Cu(OTf)	(*S*_a_)-**16b**	--	<5	--
9 ^4^	Cu(OTf)	(*S*_a_)-**16c**	--	<5	--
10 ^4^	Cu(OTf)	(*S*_a_,*R*,*R*)-**15**	--	<5	--
11	AgClO_4_	(*S*_a_,*S*,*S*)-**15**	69:31	78	−45
12	AgClO_4_	(*R*_a_,*S*,*S*)-**15**	88:12	78	94
13	AgOAc	(*R*_a_,*S*,*S*)-**15**	80:20	76	90
14 ^5^	AgOAc	(*R*_a_,*S*,*S*)-**15**	72:28	76	<30
15	AgOBz	(*R*_a_,*S*,*S*)-**15**	83:17	73	87
16	AgSbF_6_	(*R*_a_,*S*,*S*)-**15**	90:10	37	86
17	AgOTf	(*R*_a_,*S*,*S*)-**15**	87:13	75	92
18	Ag_2_CO_3_	(*R*_a_,*S*,*S*)-**15**	50:50	68	--
19	AgF	(*R*_a_,*S*,*S*)-**15**	85:15	70	82
20	AgTFA	(*R*_a_,*S*,*S*)-**15**	80:20	69	80
21 ^5^	AgClO_4_	(*R*_a_,*S*,*S*)-**15**	92:8	56	94

^1^ The ratio was determined by analysis of the ^1^H NMR spectra of the crude compound. ^2^ Chemical yields isolated after flash chromatography. ^3^ Determined by HPLC analysis using chiral stationary phase columns, for the major *endo-***9aa** diastereoisomer. ^4^ Complex with toluene. ^5^ The reaction was performed at 0 °C.

**Table 2:** Results of the brief optimization of 1,3-DC of **5a** and **8b**.

Entry	Metal Salt	Conv (%) ^2^	*endo:exo* ^1^	*Ee* ^3^
1	AgClO_4_	>95	70:30	60
2 ^4^	AgClO_4_	48	70:30	60
3 ^5^	AgClO_4_	>95	73:27	68
4	AgOTf	>95	58:42	31
5 ^6^	Ag_2_CO_3_	>95	70:30	60
6 ^4,6^	Ag_2_CO_3_	42	70:30	60
7	Ag_2_CO_3_	>95	75:25	70
8 ^7^	Ag_2_CO_3_	>95	75:25	70
9	AgTFA	>95	60:40	20
10	AgOBz	>95	47:53	29
11	AgSBF_6_	>95	60:40	55
12	AgF	>95	60:40	53
13 ^4^	Cu(OTf)	0	-	-
14 ^4^	Cu(OTf)_2_	0	-	-

^1^ Conversion was determined by analysis of the ^1^H NMR spectra of the crude compound. ^2^ The ratio was determined by analysis of the ^1^H NMR spectra of the crude compound. ^3^ Determined by HPLC analysis using chiral stationary phase columns for the major *endo-***9ba** diastereoisomer. ^4^ The reaction was performed at 0 °C. ^5^ The reaction was performed with AgClO_4_ (10 mol%) and Et_3_N (10 mol%). ^6^ The reaction was performed with AgClO_4_ (2.5 mol%). ^7^ Without triethylamine.

## Data Availability

Not applicable.

## Supplementary Materials

The following supporting information can be downloaded at: https://www.mdpi.com/article/10.3390/molecules27144579/s1.

## Sample Availability

Samples of all of the compounds described are available from the authors.
