# Supplementary material for: Enantioselective 1,3-Dipolar Cycloaddition Using (Z)-α-Amidonitroalkenes as a Key Step to the Access to Chiral cis-3,4-Diaminopyrrolidines
Source: Molecules. 2022 Jul 18;27(14):4579. doi: 10.3390/molecules27144579 (PMC9316397; doi:10.3390/molecules27144579)
Supplement: Supplementary file 1 [file molecules-27-04579-s001.zip › molecules-1819254-SI.pdf]

Supplementary Materials

Enantioselective 1,3-Dipolar Cycloaddition Using (*Z*)-α-Amidonitroalkenes as a Key Step to the Access to Chiral *cis*-3,4-Diaminopyrrolidines †

Eduardo García-Mingüens [1,2], Marcos Ferrándiz-Saperas [1,2], M. de Gracia Retamosa [1,2], Carmen Nájera [2,*], Miguel Yus [2] and José M. Sansano [1,2,*]

1 Instituto de Síntesis Orgánica (ISO), Centro de Innovación en Química Avanzada (ORFEO-CINQA),
Departamento de Química Orgánica, University of Alicante, P.O. Box 99, 03690 San Vicente del Raspeig, Spain; eduardo.gm@ua.es (E.G.-M.); marcos.ferrandiz@ua.es (M.F.-S.); gracia.retamosa@ua.es (M.d.G.R.)
2 Centro de Innovación en Química Avanzada (ORFEO-CINQA), P.O. Box 99, 03690 San Vicente del Raspeig, Spain; yus@ua.es
* Correspondence: cnajera@ua.es (C.N.); jmsansano@ua.es (J.M.S.); Tel.: +34-965-90-3549 (J.M.S.)
† Dedicated to Prof. Henri B. Kagan.

Table of contents:

1. NMR Spectra of Products ………………………………………………………… S2
2. HPLC Chromatograms ………………………………………………………………… S16

1. NMR spectra of Products

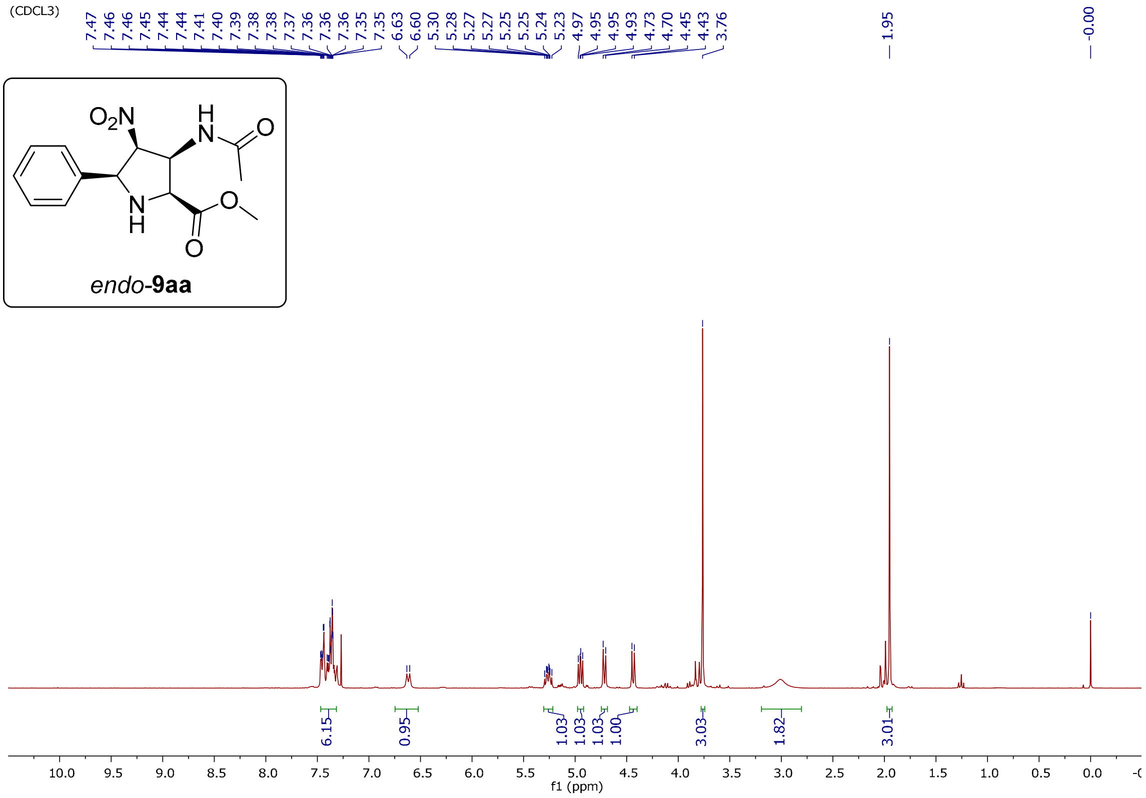
(CDCL3)
7.47 7.46 7.46 7.45 7.44 7.44 7.41 7.40 7.39 7.38 7.38 7.37 7.36 7.36 7.36 7.35 6.63 6.60 5.30 5.28 5.27 5.27 5.25 5.25 5.24 5.23 4.97 4.95 4.95 4.93 4.73 4.70 4.45 4.43 3.76
1.95
-0.00
O_2N
H
N
O
N
H
O
O
endo-**9aa**
6.15
0.95
1.03
1.03
1.03
1.00
3.03
1.82
3.01
10.0 9.5 9.0 8.5 8.0 7.5 7.0 6.5 6.0 5.5 5.0 4.5 4.0 3.5 3.0 2.5 2.0 1.5 1.0 0.5 0.0
f1 (ppm)

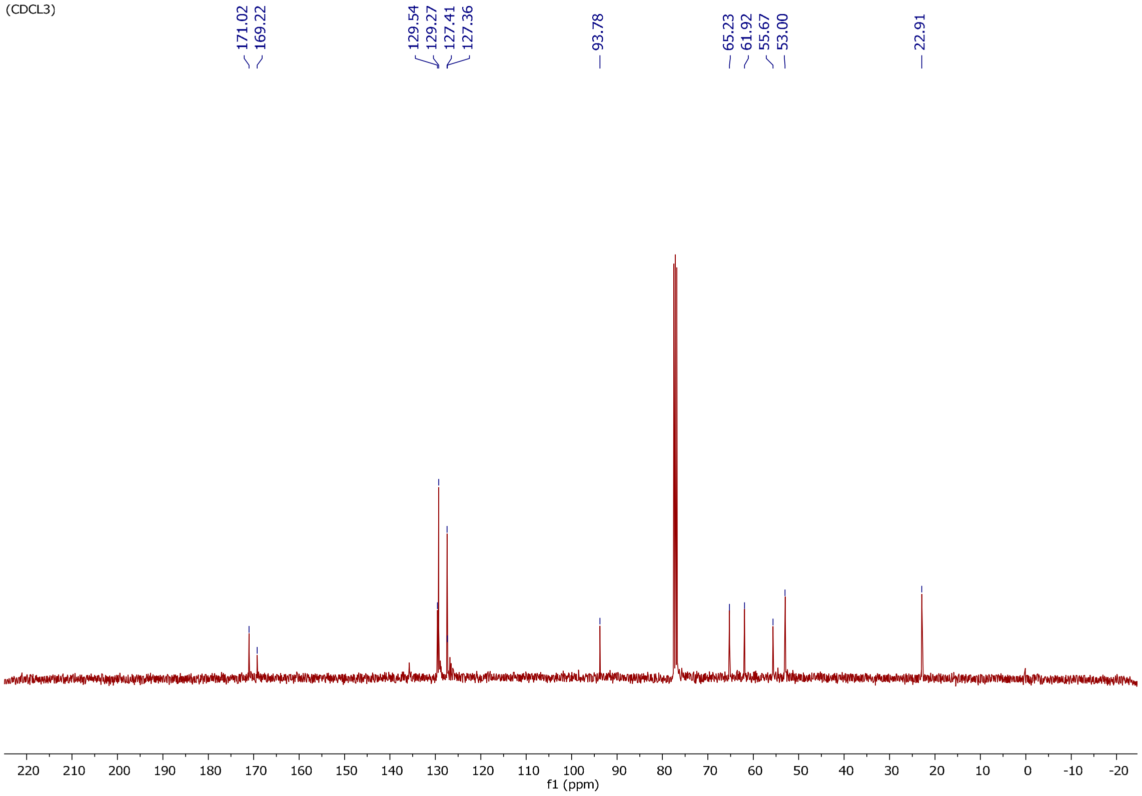
(CDCL3)
171.02 169.22
129.54 129.27 127.41 127.36
93.78
65.23 61.92 55.67 53.00
22.91
220 210 200 190 180 170 160 150 140 130 120 110 100 90 80 70 60 50 40 30 20 10 0 -10 -20
f1 (ppm)

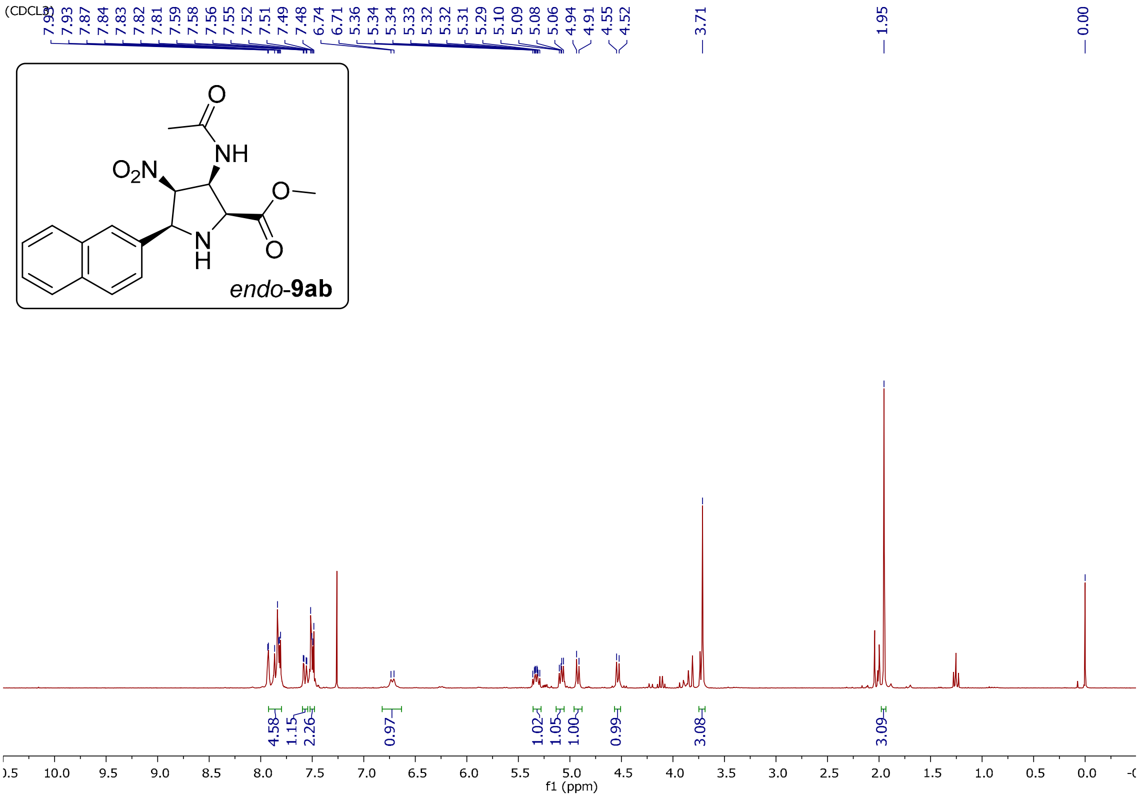
(CDCL3)
7.93 7.87 7.84 7.83 7.82 7.81 7.59 7.58 7.56 7.55 7.52 7.51 7.49 7.48 6.74 6.71 5.36 5.34 5.34 5.33 5.32 5.32 5.31 5.29 5.10 5.09 5.08 5.06 4.94 4.91 4.55 4.52 3.71 1.95 0.00
O
NH
O_2N
O
N
H
O
endo-**9ab**
4.58 1.15 2.26 0.97 1.02 1.05 1.00 0.99 3.08 3.09
f1 (ppm)

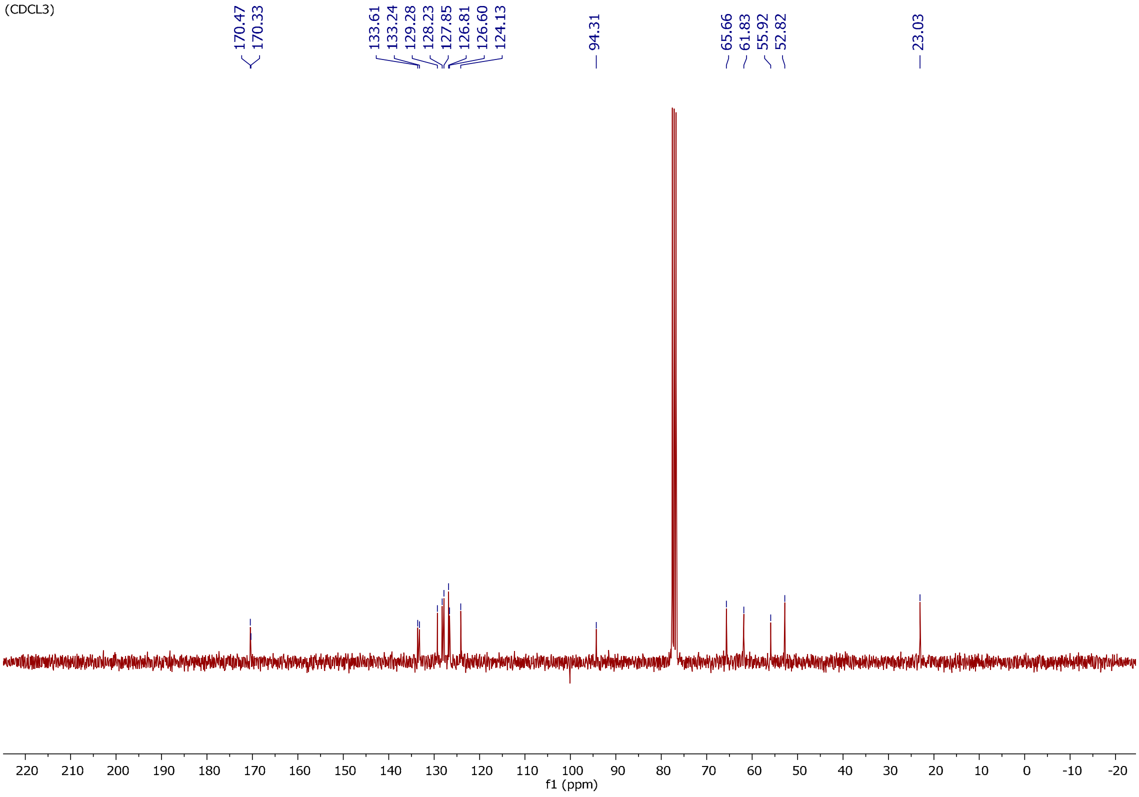
(CDCL3)
170.47 170.33 133.61 133.24 129.28 128.23 127.85 126.81 126.60 124.13 94.31 65.66 61.83 55.92 52.82 23.03
f1 (ppm)

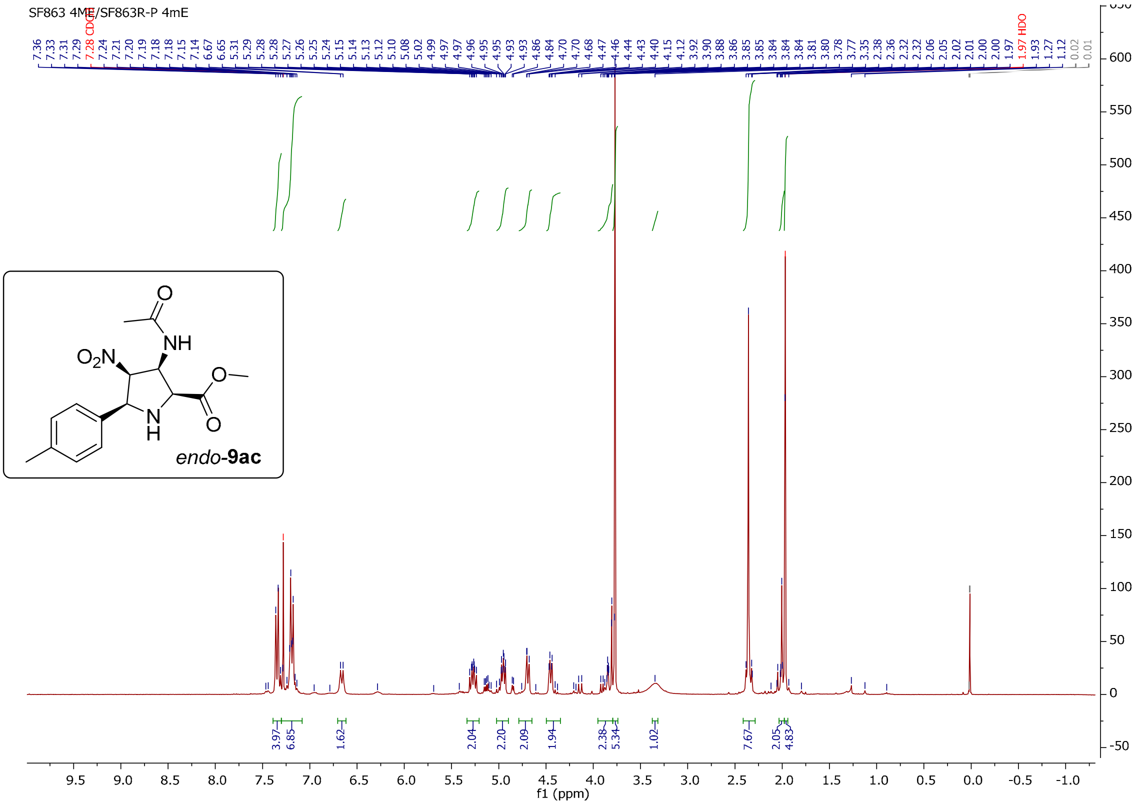
SF863 4ME/SF863R-P 4mE
7.36 7.33 7.31 7.29 7.28 CDCl3 7.24 7.21 7.20 7.19 7.18 7.18 7.15 7.14 6.67 6.65 5.31 5.29 5.28 5.28 5.27 5.26 5.25 5.24 5.15 5.14 5.13 5.12 5.10 5.08 5.02 4.99 4.97 4.97 4.96 4.95 4.95 4.93 4.93 4.86 4.84 4.70 4.68 4.47 4.46 4.44 4.43 4.40 4.15 4.12 3.92 3.90 3.88 3.86 3.85 3.85 3.84 3.84 3.84 3.81 3.80 3.78 3.77 3.35 2.38 2.36 2.32 2.32 2.06 2.05 2.02 2.01 2.00 2.00 1.97 1.97 HDO 1.93 1.27 1.12 0.02 0.01
O
NH
O_2N
O
N
H
O
endo-**9ac**
3.97 6.85 1.62 2.04 2.20 2.09 1.94 2.38 5.34 1.02 7.67 2.05 4.83
f1 (ppm)

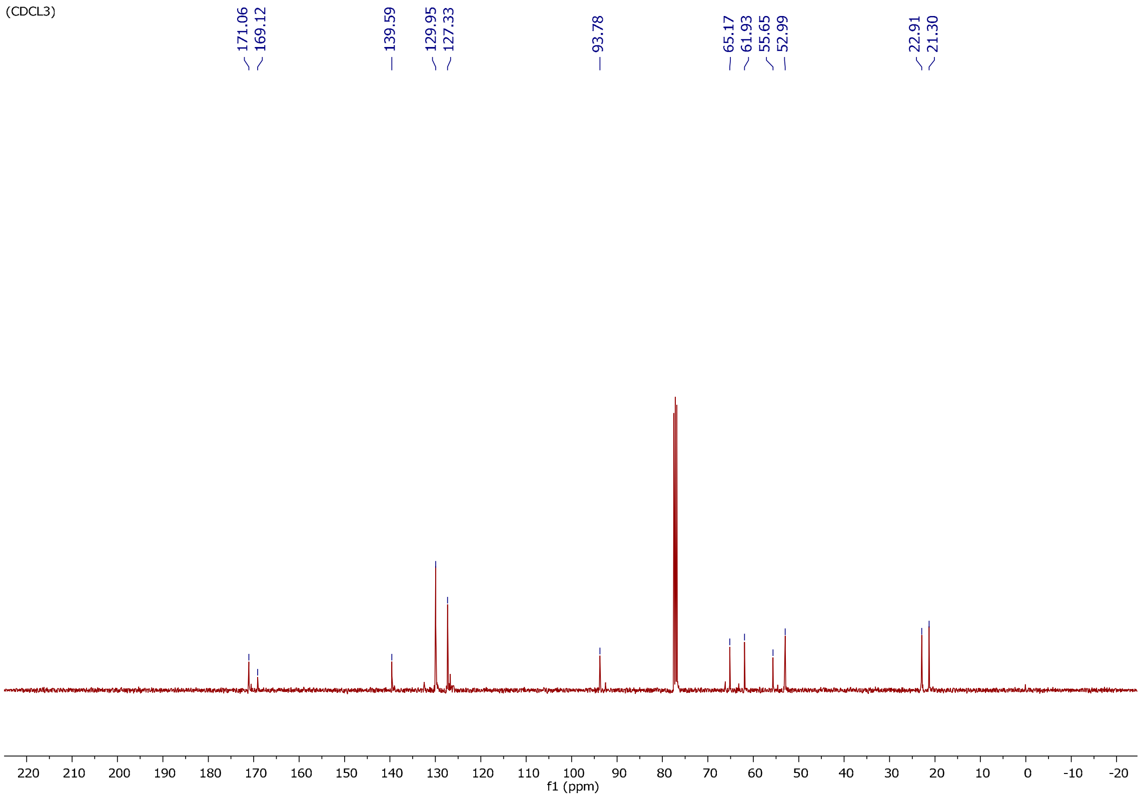
(CDCL3)
171.06 169.12 139.59 129.95 127.33 93.78 65.17 61.93 55.65 52.99 22.91 21.30
f1 (ppm)

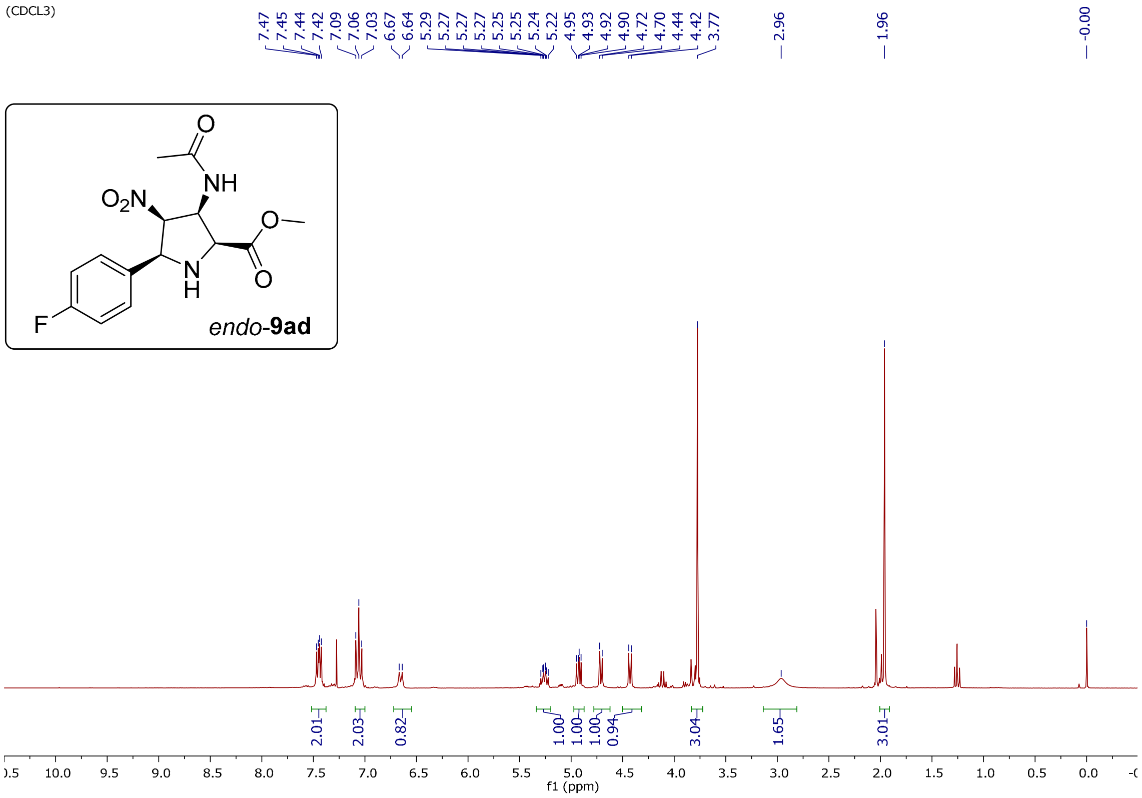
(CDCL3)
7.47
7.45
7.44
7.42
7.09
7.06
7.03
6.67
6.64
5.29
5.27
5.27
5.27
5.25
5.25
5.24
5.22
4.95
4.93
4.92
4.90
4.72
4.70
4.44
4.42
3.77
2.96
1.96
-0.00
O
NH
O_2N
O
N
H
O
F
endo-**9ad**
2.01
2.03
0.82
1.00
1.00
1.00
0.94
3.04
1.65
3.01
f1 (ppm)

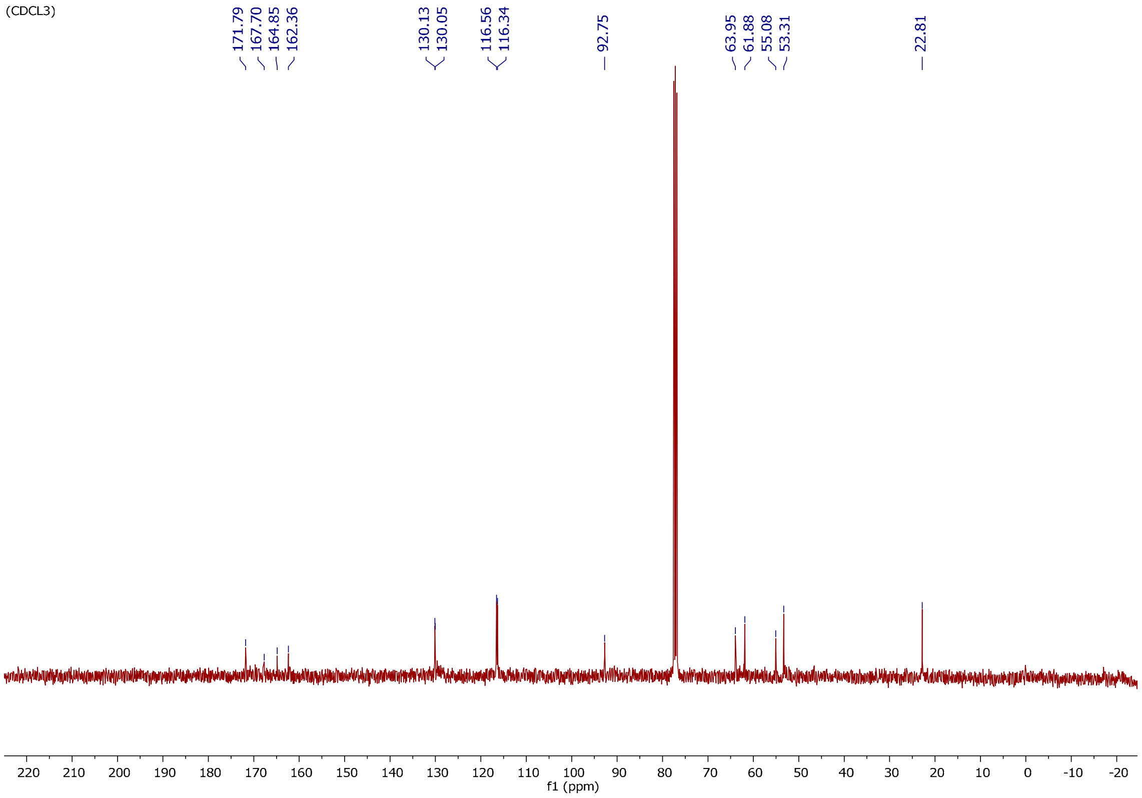
(CDCL3)
171.79
167.70
164.85
162.36
130.13
130.05
116.56
116.34
92.75
63.95
61.88
55.08
53.31
22.81
f1 (ppm)

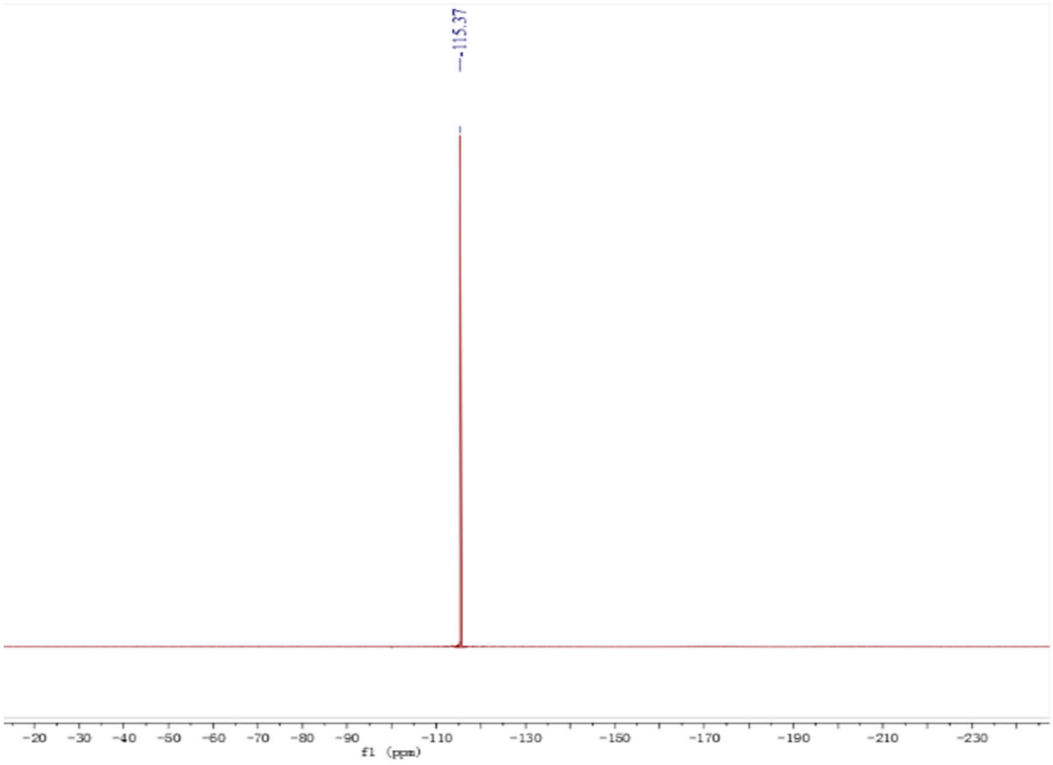

-115.37
-20
-30
-40
-50
-60
-70
-80
-90
-110
-130
-150
-170
-190
-210
-230
f1 (ppm)

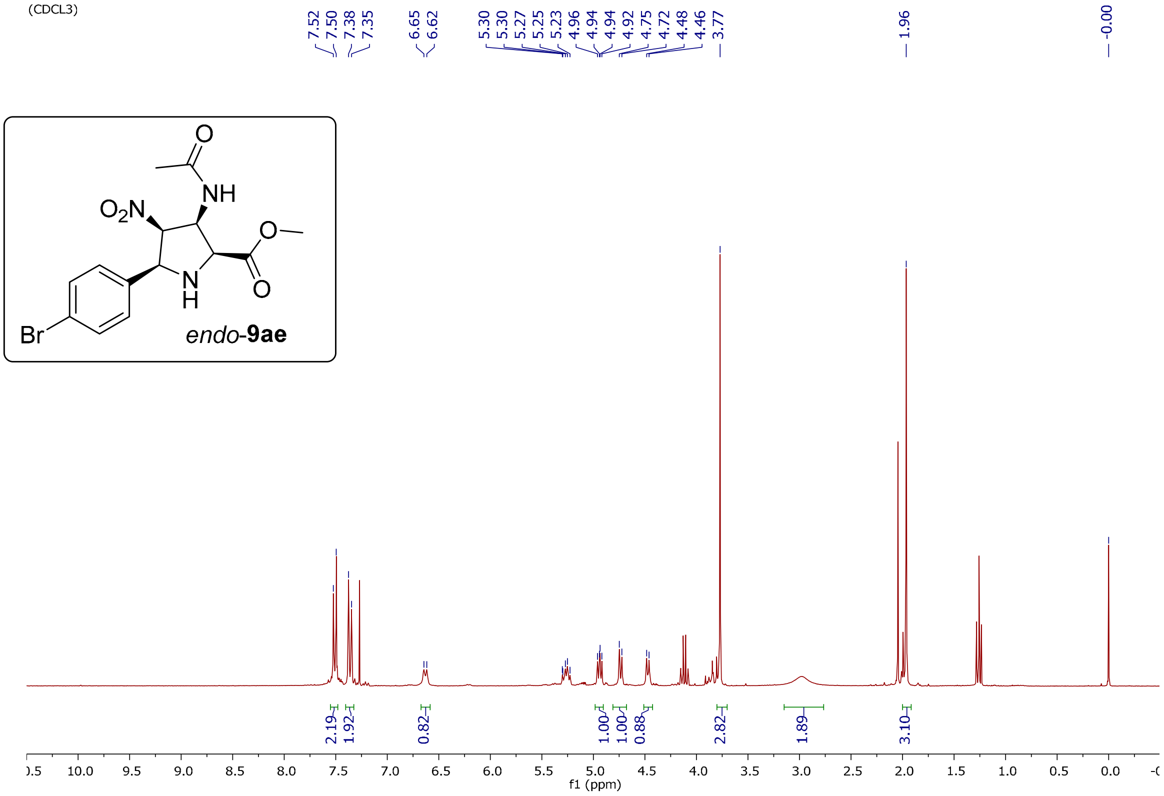
(CDCL3)
7.52 7.50 7.38 7.35 6.65 6.62 5.30 5.30 5.27 5.25 5.23 4.96 4.94 4.94 4.92 4.75 4.72 4.48 4.46 3.77 1.96 -0.00
O_2N
NH
O
N
H
Br
endo-**9ae**
2.19 1.92 0.82 1.00 1.00 0.88 2.82 1.89 3.10
f1 (ppm)

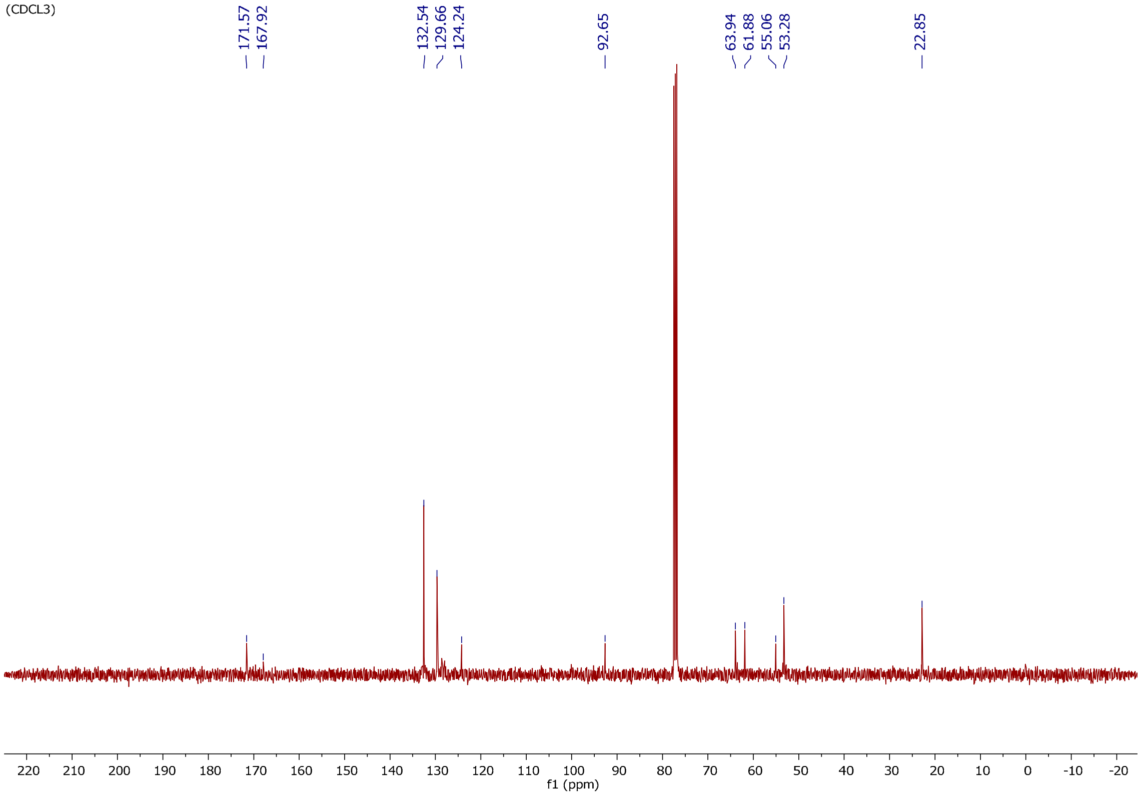
(CDCL3)
171.57 167.92 132.54 129.66 124.24 92.65 63.94 61.88 55.06 53.28 22.85
f1 (ppm)

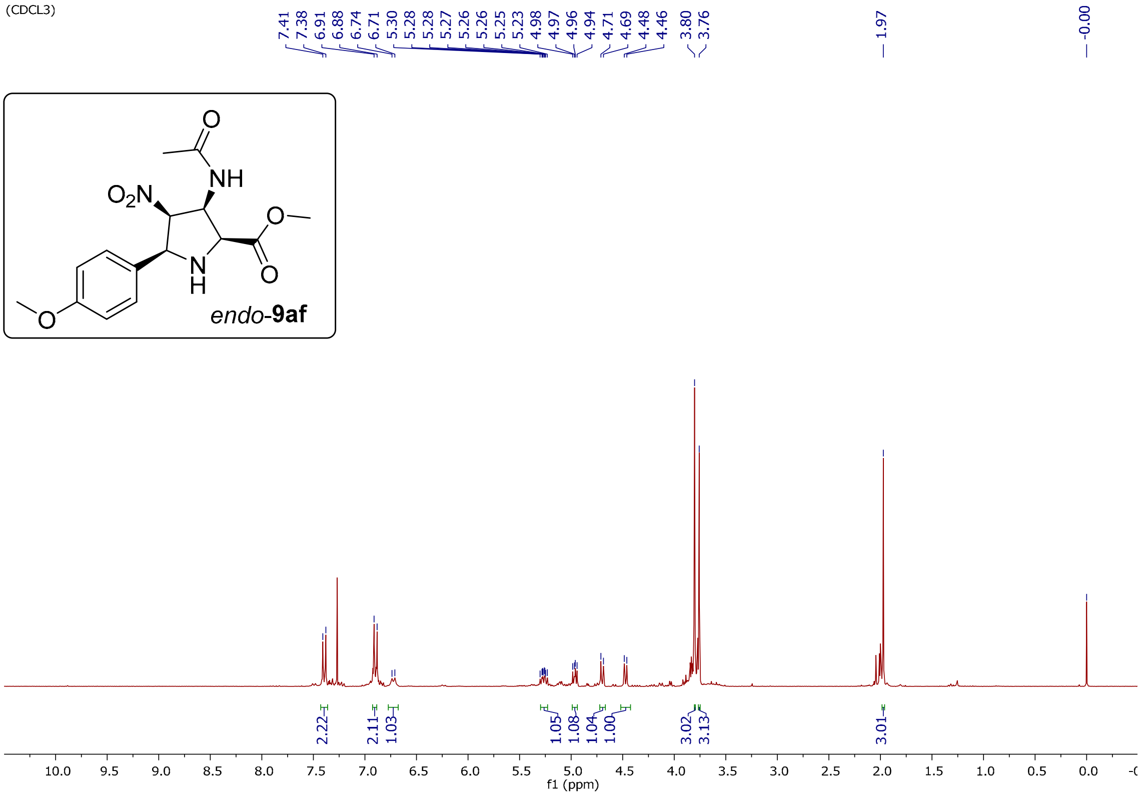
(CDCL3)
7.41 7.38 6.91 6.88 6.74 6.71 5.30 5.28 5.28 5.27 5.26 5.26 5.25 5.23 4.98 4.97 4.96 4.94 4.71 4.69 4.48 4.46 3.80 3.76
1.97
-0.00
O
NH
O2N
O
N
H
O
O
endo-9af
2.22 2.11 1.03 1.05 1.08 1.04 1.00 3.02 3.13 3.01
10.0 9.5 9.0 8.5 8.0 7.5 7.0 6.5 6.0 5.5 5.0 4.5 4.0 3.5 3.0 2.5 2.0 1.5 1.0 0.5 0.0
f1 (ppm)

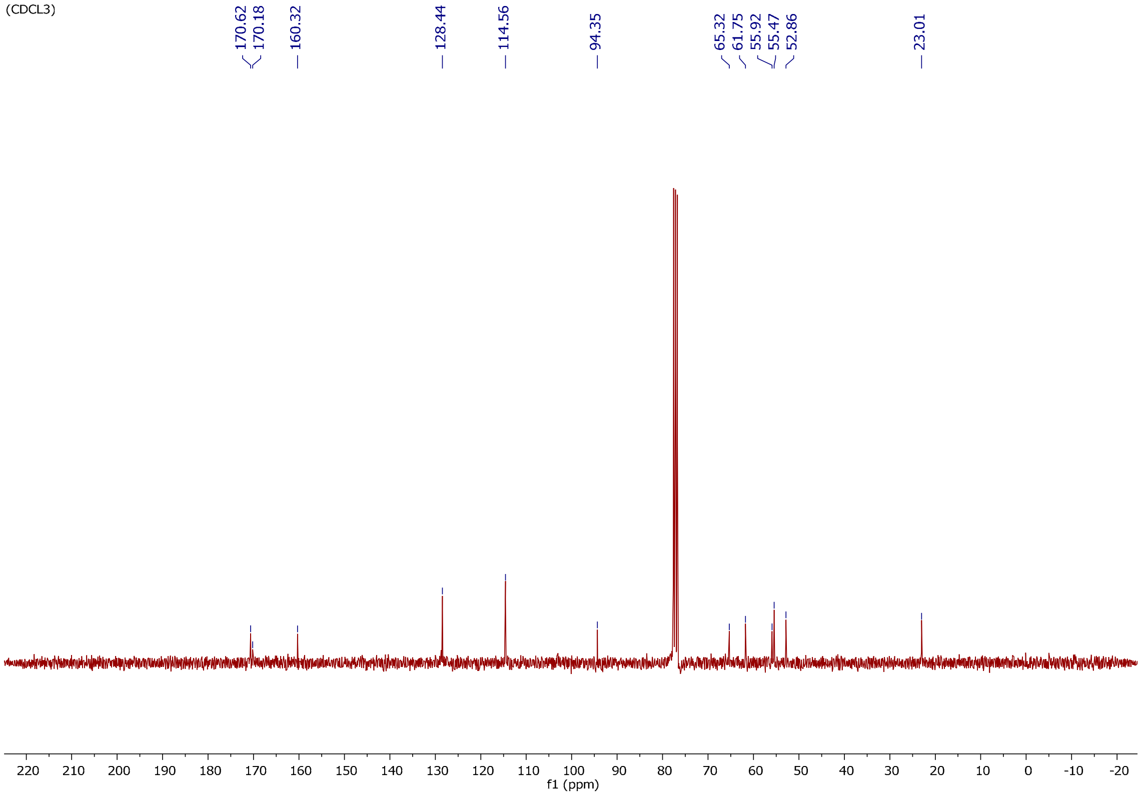
(CDCL3)
170.62 170.18 160.32
128.44
114.56
94.35
65.32 61.75 55.92 55.47 52.86
23.01
220 210 200 190 180 170 160 150 140 130 120 110 100 90 80 70 60 50 40 30 20 10 0 -10 -20
f1 (ppm)

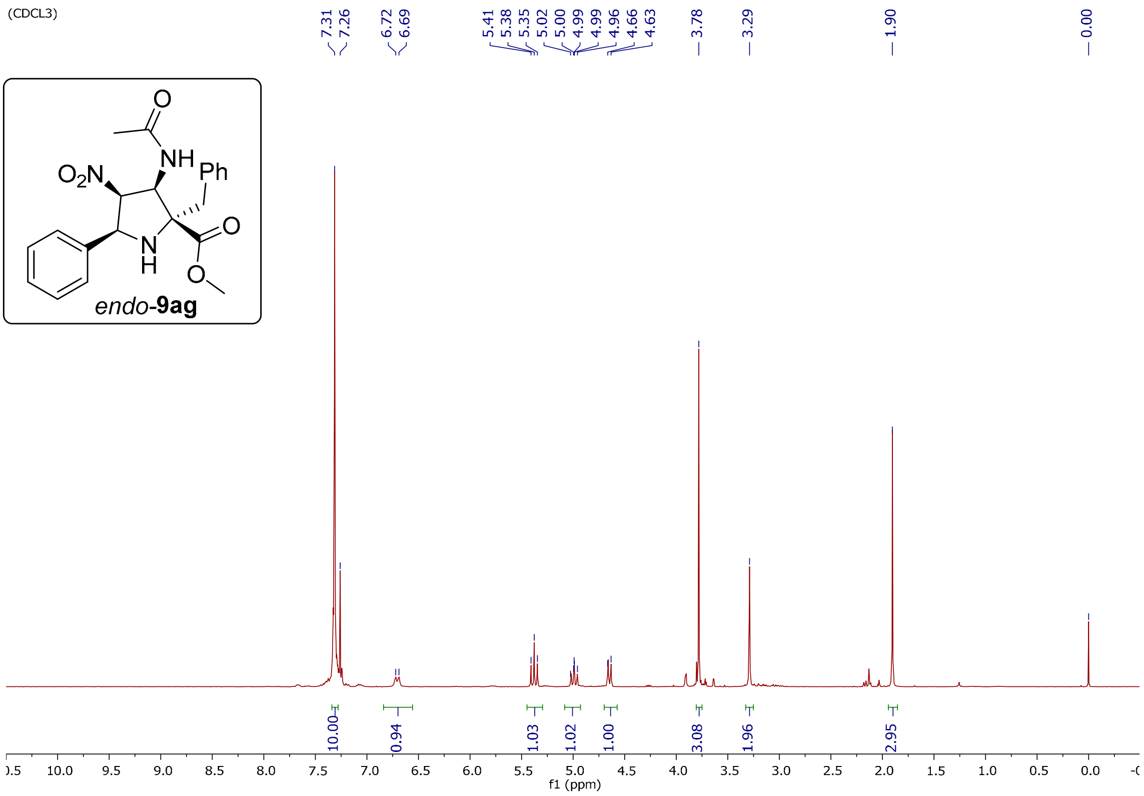
(CDCL3)
7.31
7.26
6.72
6.69
5.41
5.38
5.35
5.02
5.00
4.99
4.99
4.96
4.66
4.63
3.78
3.29
1.90
0.00
O
NH
Ph
O2N
O
N
H
O
endo-9ag
10.00
0.94
1.03
1.02
1.00
3.08
1.96
2.95
f1 (ppm)

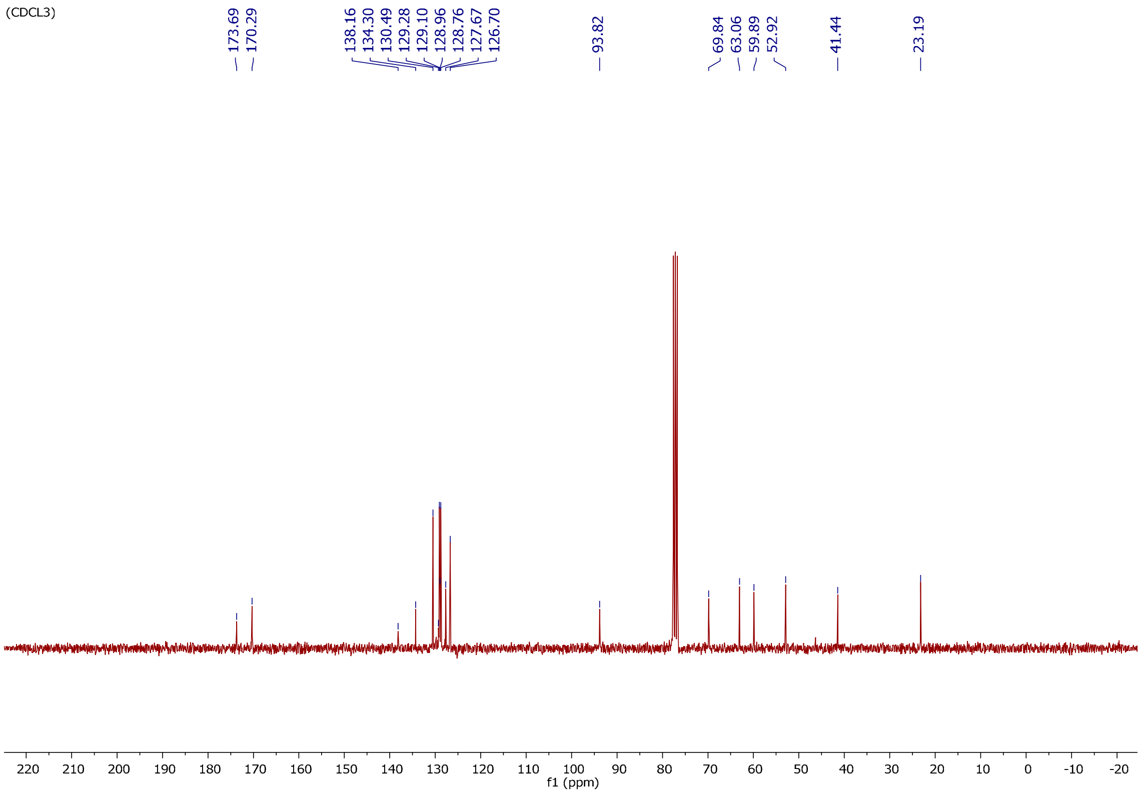
(CDCL3)
173.69
170.29
138.16
134.30
130.49
129.28
129.10
128.96
128.76
127.67
126.70
93.82
69.84
63.06
59.89
52.92
41.44
23.19
f1 (ppm)

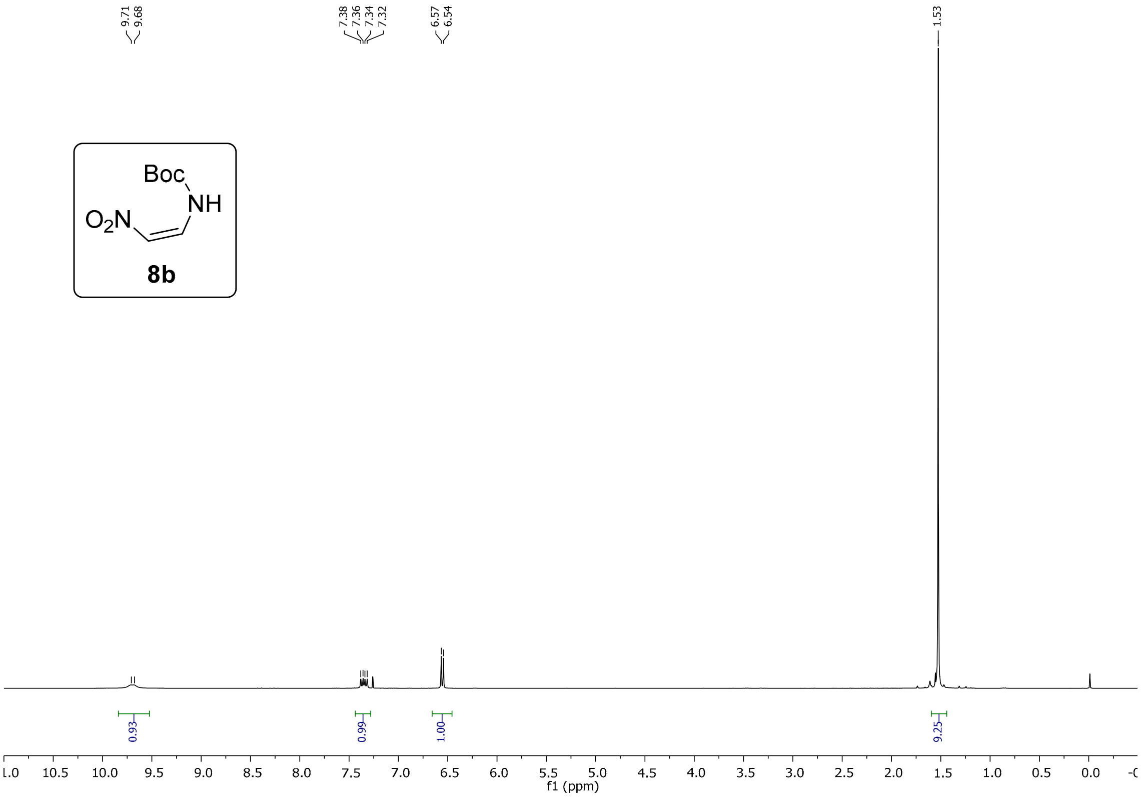
9.71
9.68
7.38
7.36
7.34
7.32
6.57
6.54
1.53
Boc
NH
O2N
8b
0.93
0.99
1.00
9.25
f1 (ppm)

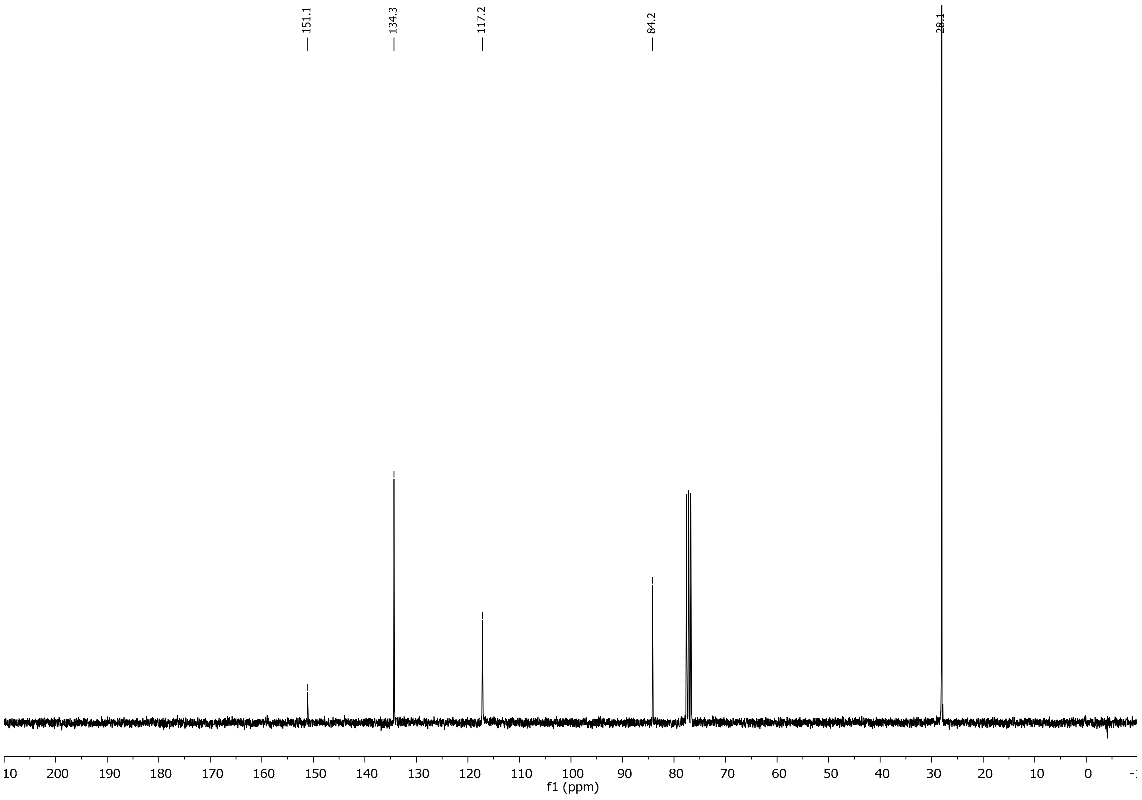
151.1
134.3
117.2
84.2
28.1
f1 (ppm)

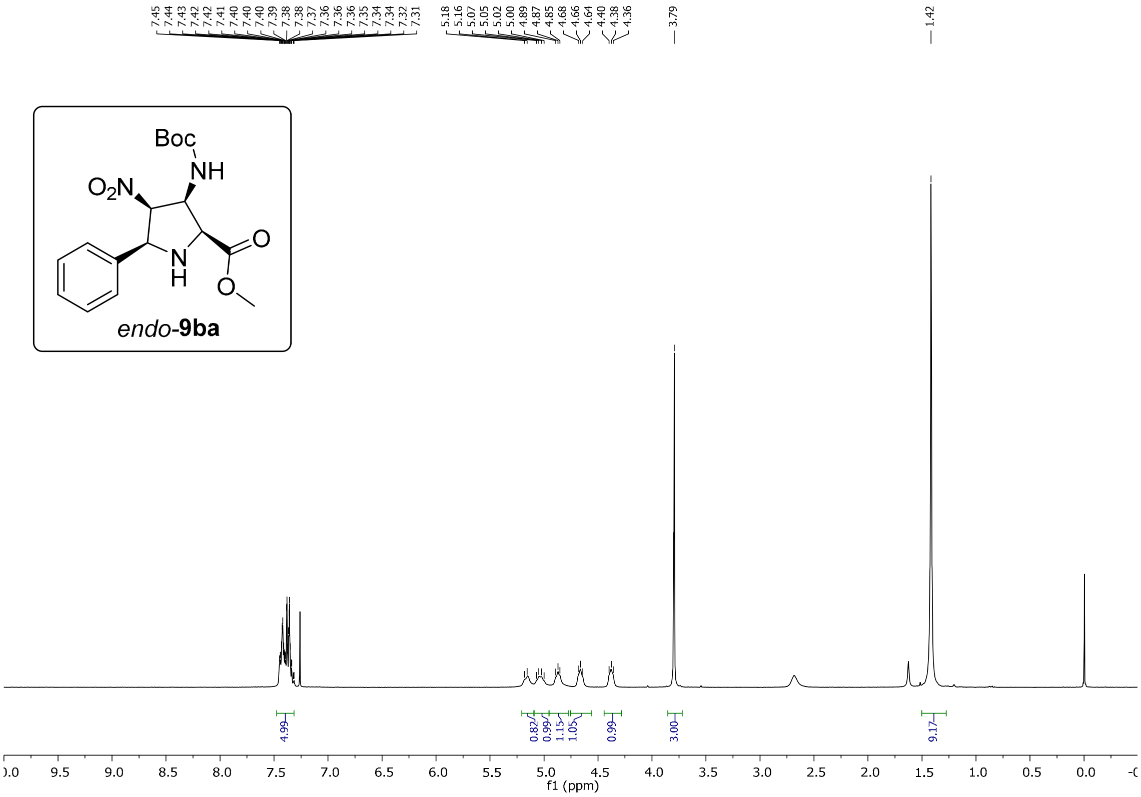
7.45 7.44 7.43 7.42 7.42 7.41 7.40 7.40 7.40 7.39 7.38 7.38 7.37 7.36 7.36 7.35 7.34 7.34 7.32 7.31
5.18 5.16 5.07 5.05 5.02 5.00 4.89 4.87 4.85 4.68 4.66 4.64 4.40 4.38 4.36
3.79
1.42
Boc
NH
O2N
N
H
O
endo-9ba
4.99
0.82
0.99
1.15
1.05
0.99
3.00
9.17
f1 (ppm)

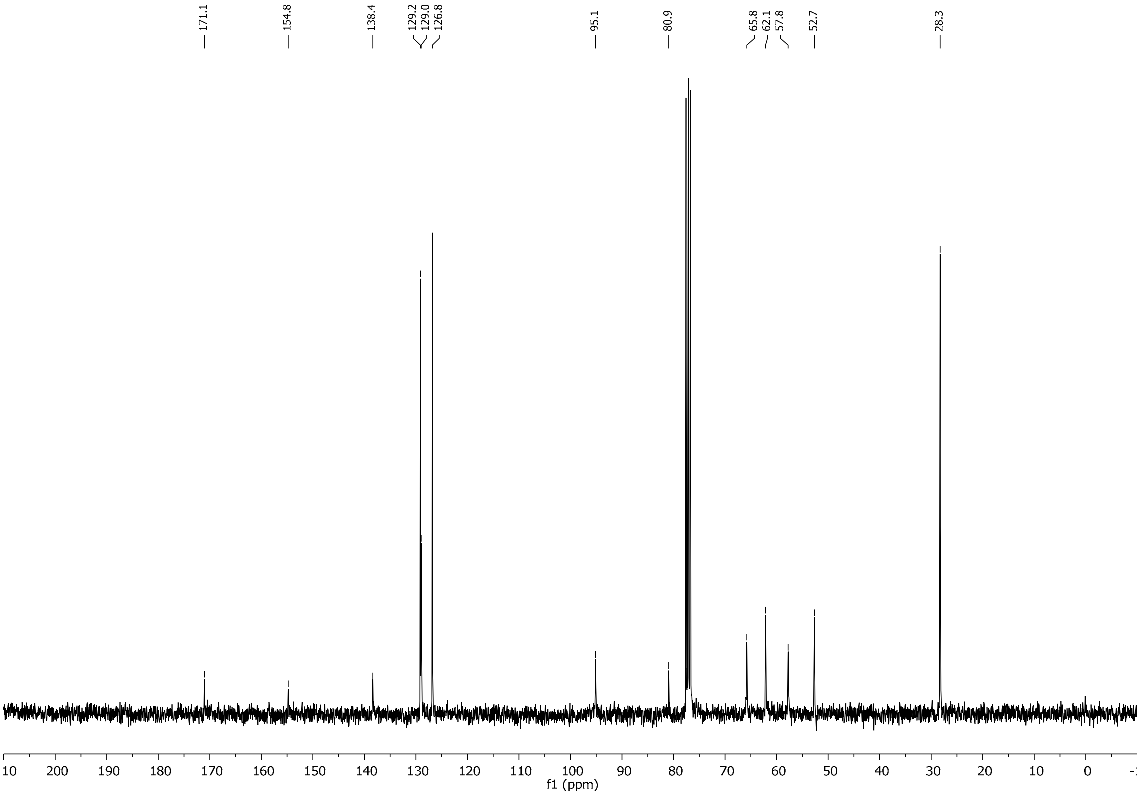
171.1
154.8
138.4
129.2 129.0 126.8
95.1
80.9
65.8 62.1 57.8
52.7
28.3
f1 (ppm)

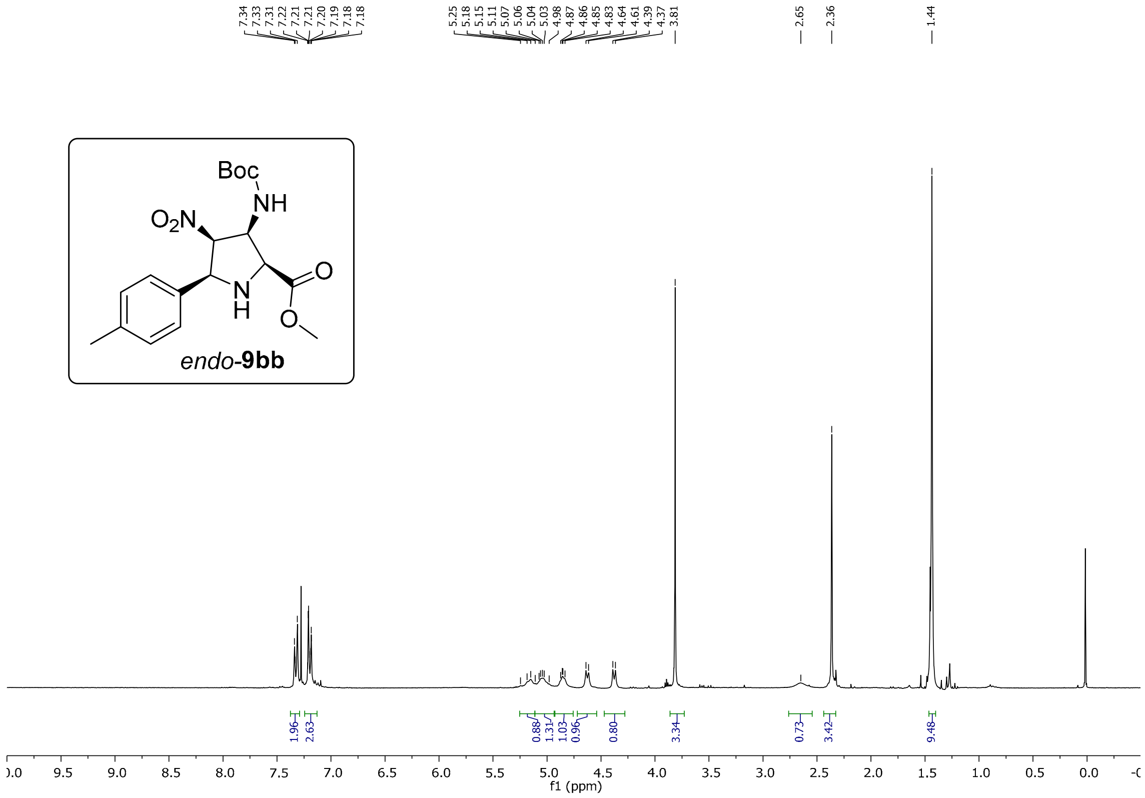
7.34 7.33 7.31 7.22 7.21 7.21 7.20 7.19 7.18 7.18
5.25 5.18 5.15 5.11 5.07 5.06 5.04 5.03 4.98 4.87 4.86 4.85 4.83 4.64 4.61 4.39 4.37 3.81
2.65 2.36
1.44
Boc
NH
O_2N
N
H
O
O
endo-**9bb**
1.96 2.63 0.88 1.31 1.03 0.96 0.80 3.34 0.73 3.42 9.48
f1 (ppm)

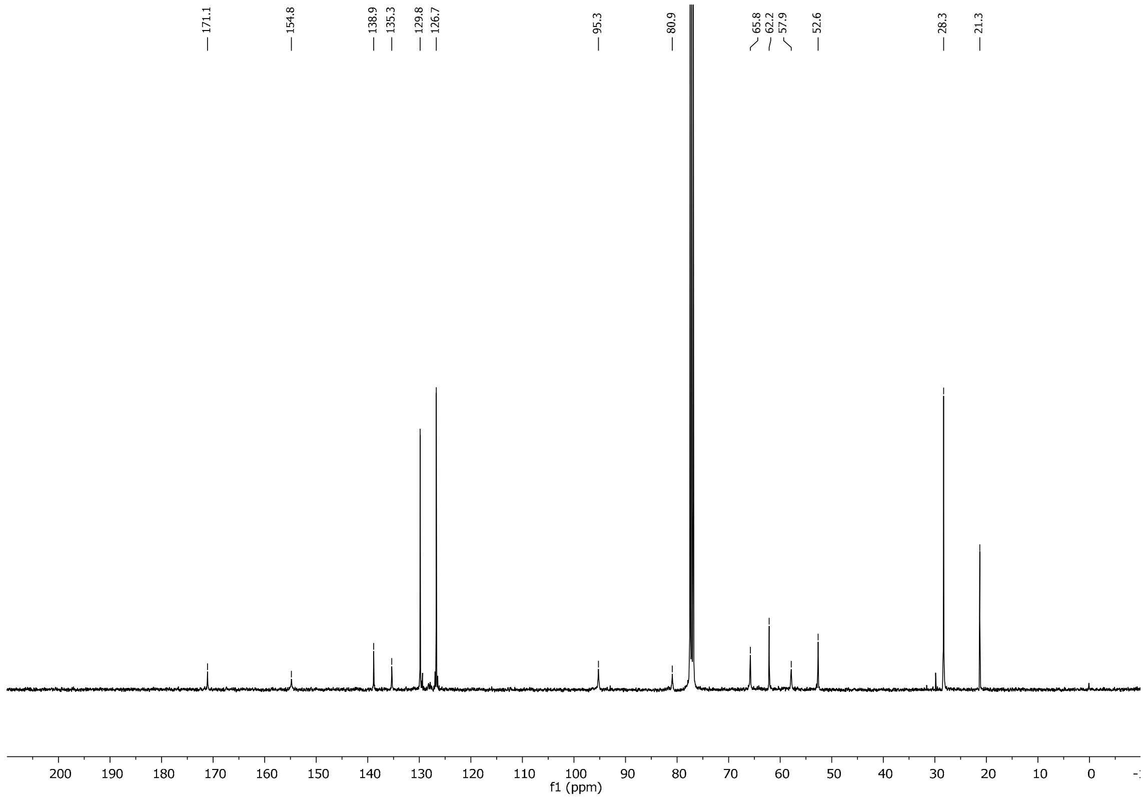
171.1 154.8 138.9 135.3 129.8 126.7 95.3 80.9 65.8 62.2 57.9 52.6 28.3 21.3
f1 (ppm)

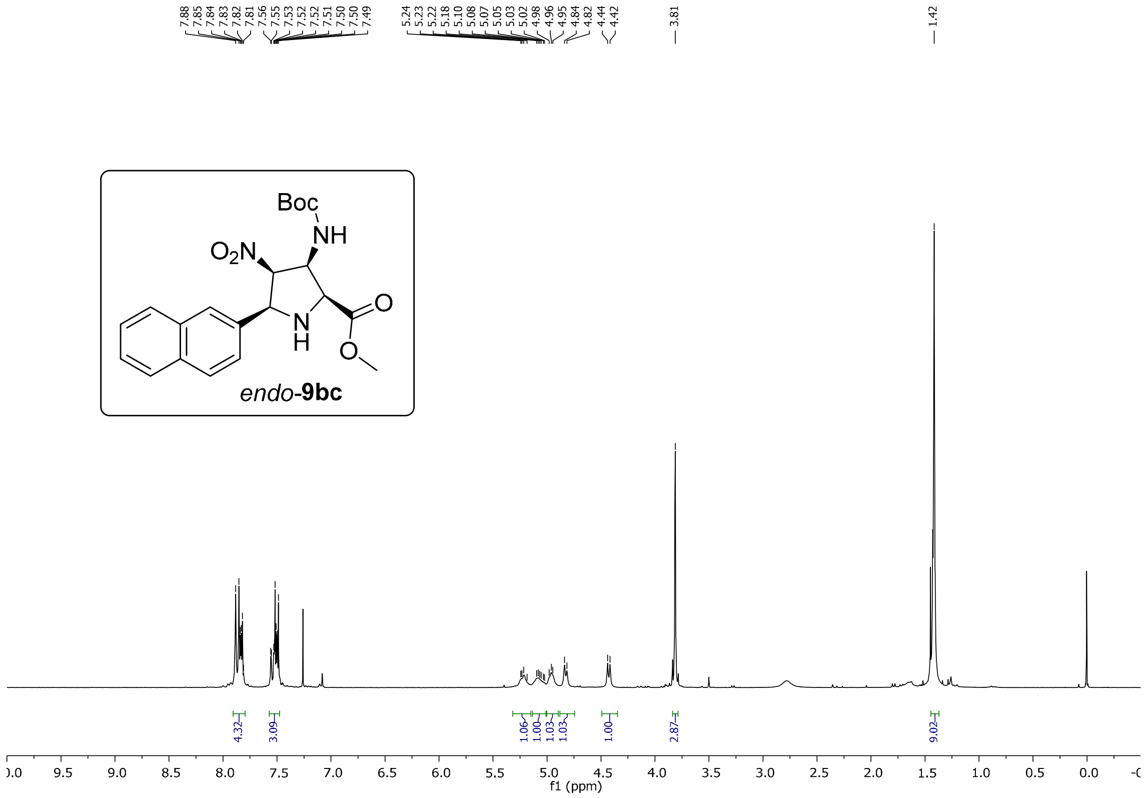
7.88 7.85 7.84 7.83 7.82 7.81 7.56 7.55 7.53 7.52 7.52 7.51 7.50 7.50 7.49
5.24 5.23 5.22 5.18 5.10 5.08 5.07 5.05 5.03 5.02 4.98 4.96 4.95 4.84 4.82 4.44 4.42
3.81
1.42
Boc
NH
O_2N
N
H
O
O
endo-**9bc**
4.32
3.09
1.06
1.00
1.03
1.03
1.00
2.87
9.02
f1 (ppm)

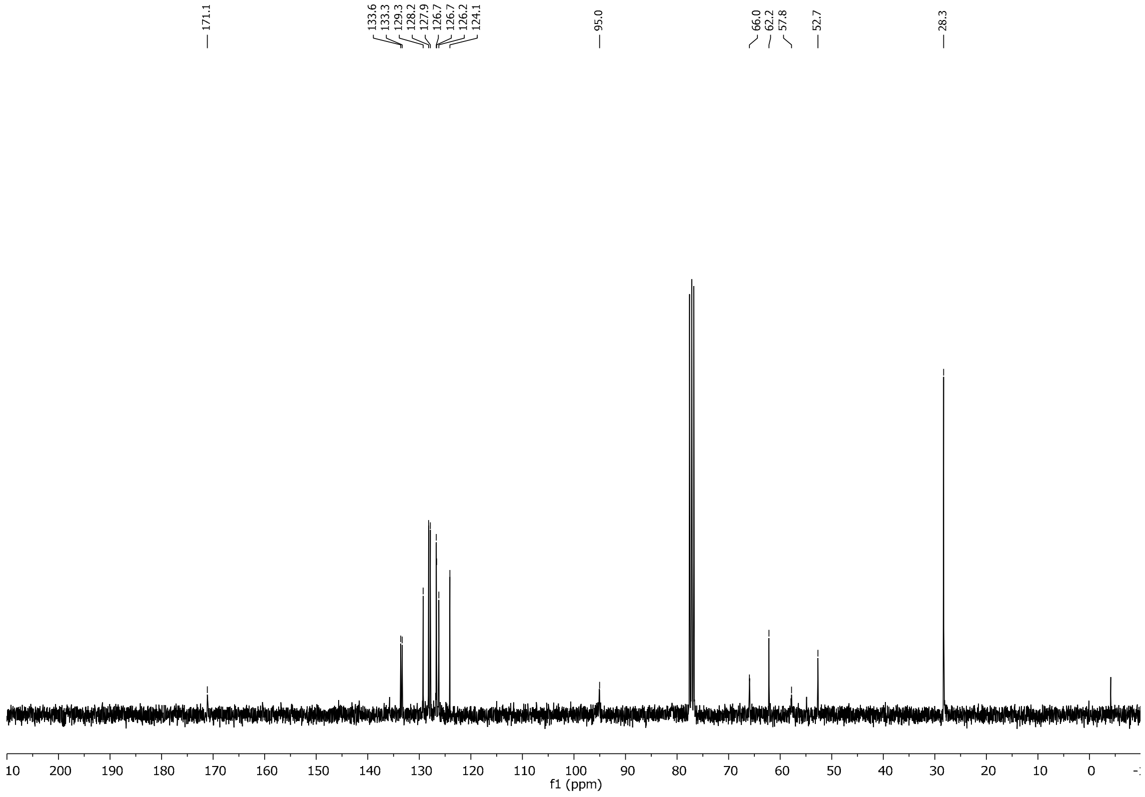
171.1
133.6 133.3 129.3 128.2 127.9 126.7 126.7 126.2 124.1
95.0
66.0 62.2 57.8
52.7
28.3
f1 (ppm)

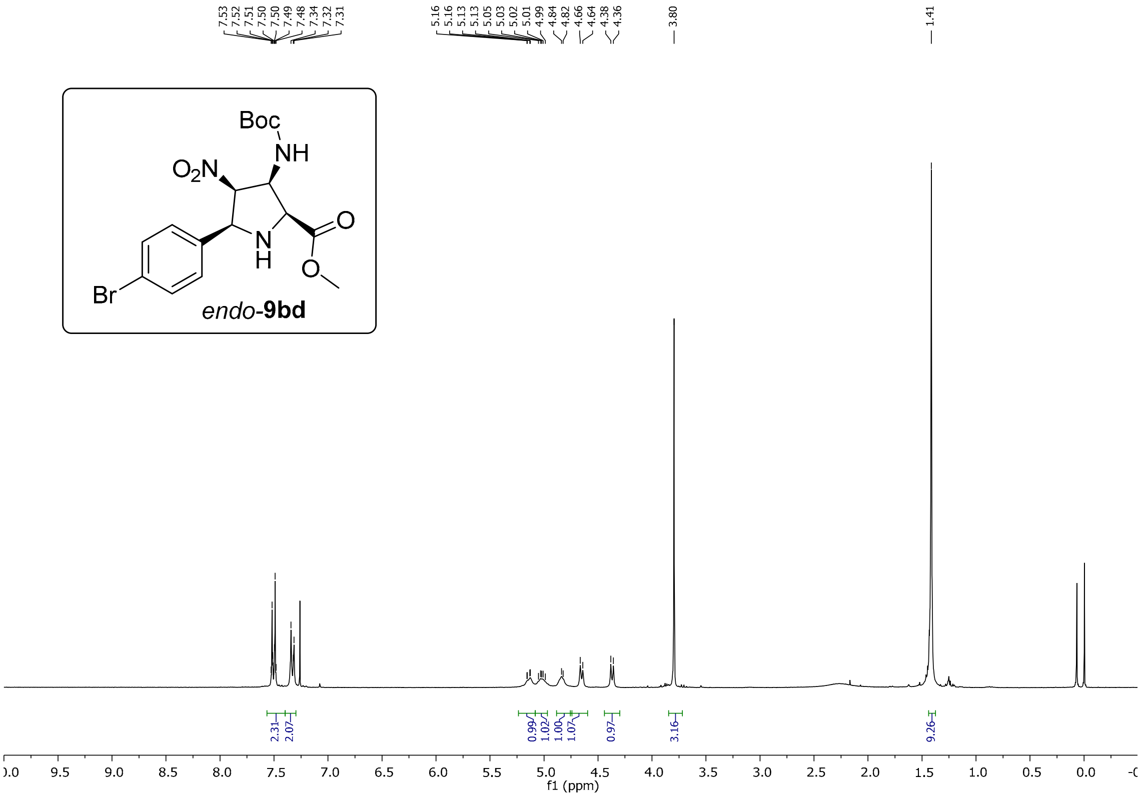
7.53 7.52 7.51 7.50 7.50 7.49 7.48 7.34 7.32 7.31
5.16 5.16 5.13 5.13 5.05 5.03 5.02 5.01 4.99 4.84 4.82 4.66 4.64 4.38 4.36
3.80
1.41
Boc
NH
O_2N
N
H
O
O
Br
endo-**9bd**
2.31 2.07 0.99 1.02 1.00 1.07 0.97 3.16 9.26
f1 (ppm)

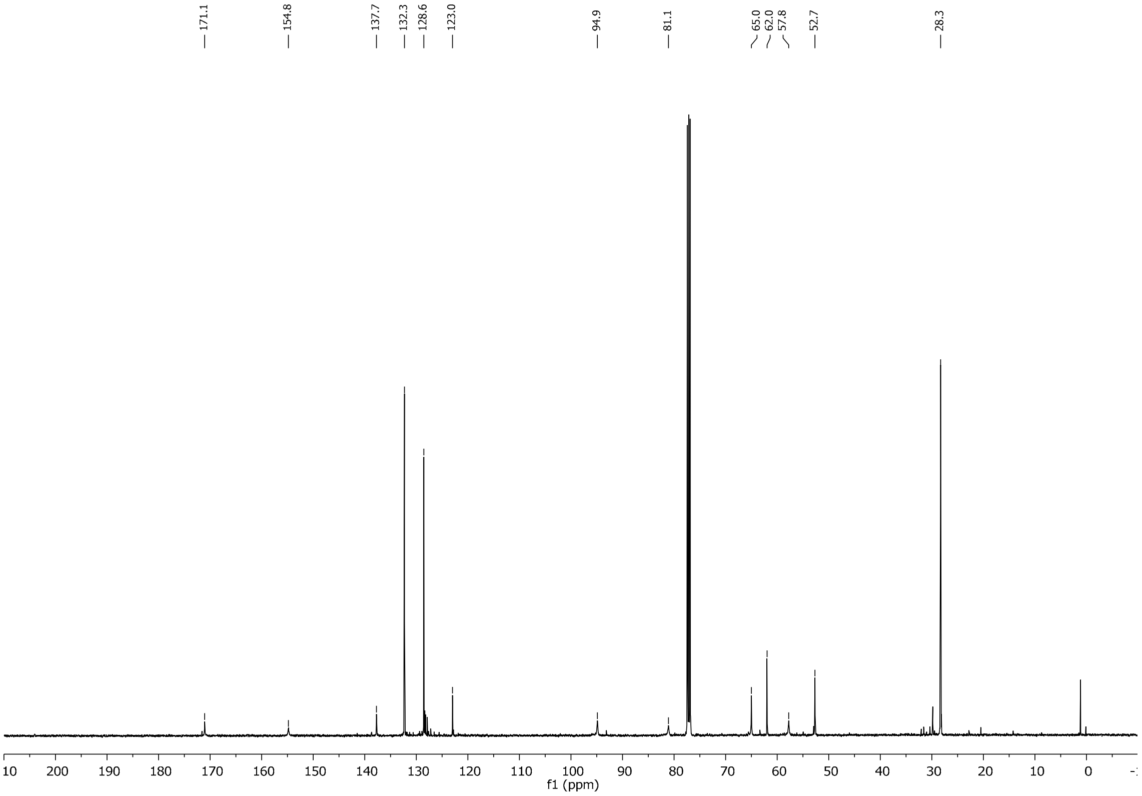
171.1
154.8
137.7 132.3 128.6 123.0
94.9
81.1
65.0 62.0 57.8 52.7
28.3
f1 (ppm)

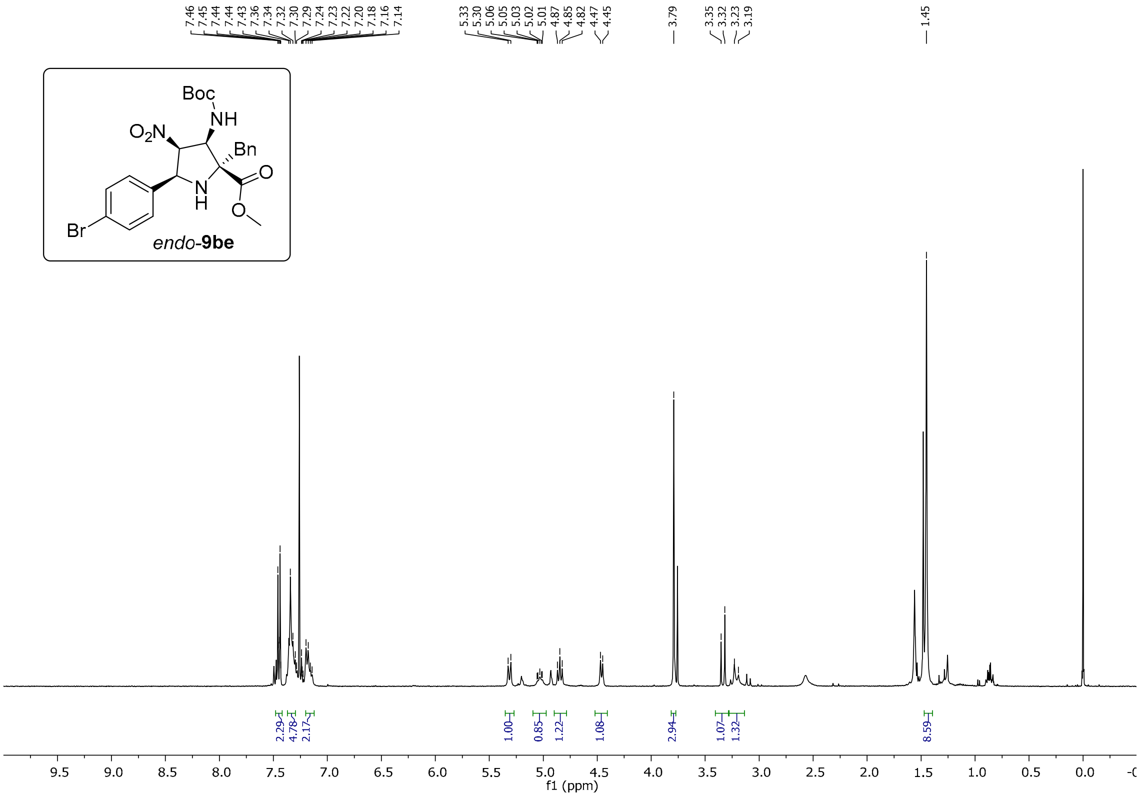
Boc
NH
O2N
Bn
O
N
H
O
Br
endo-9be
f1 (ppm)

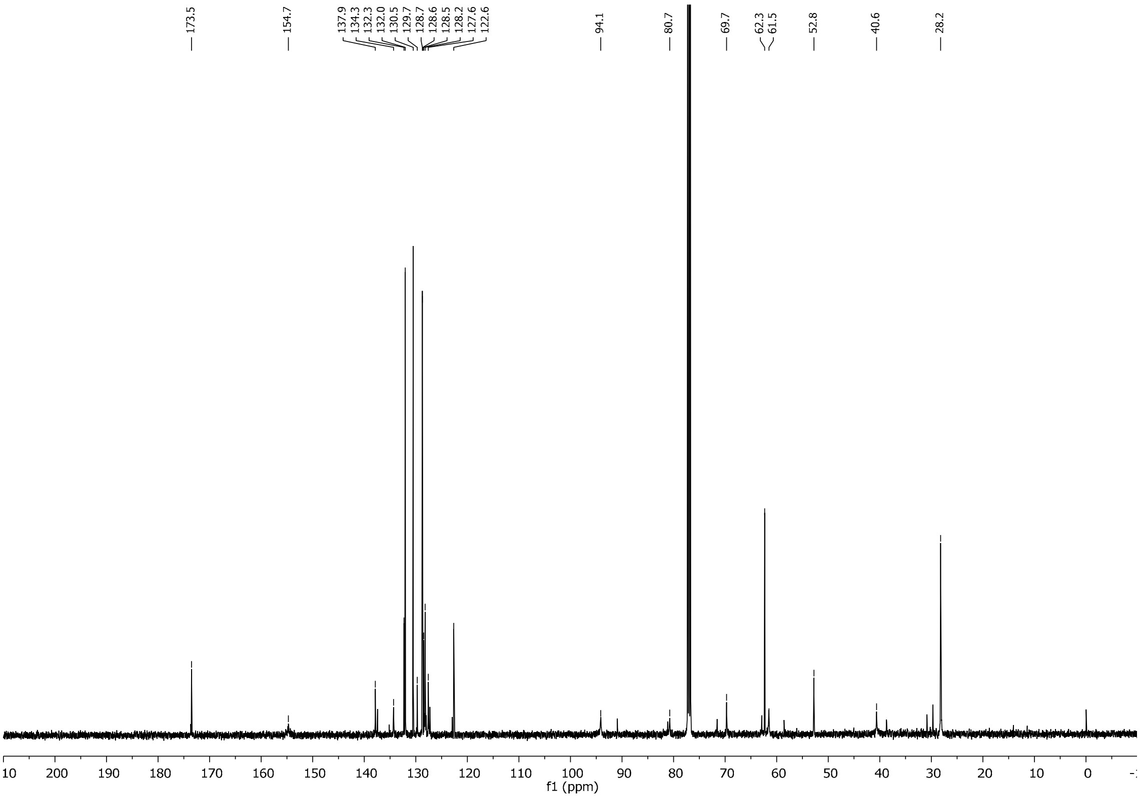
f1 (ppm)

2. HPLC Chromatograms

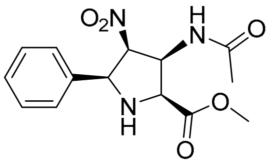

endo-**9aa**

Obtained using $AgClO_4$·(R_a,*S*,*S*)-**15**

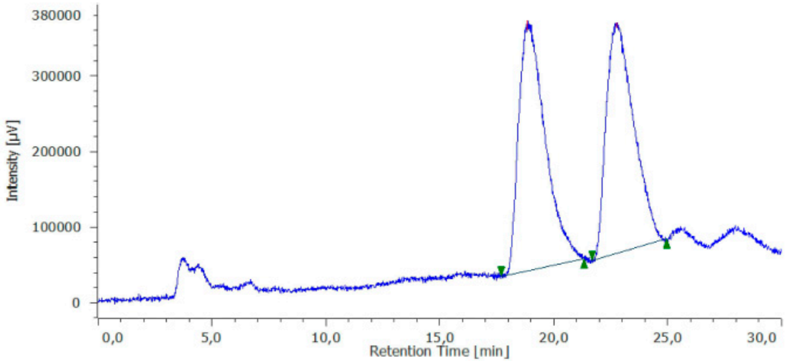

#	Peak Name	CH	tR [min]	Area [µV·sec]	Height [µV]	Area%
1	Unknown	9	18,853	26648375	330323	51,127
2	Unknown	9	22,773	25473651	310092	48,873

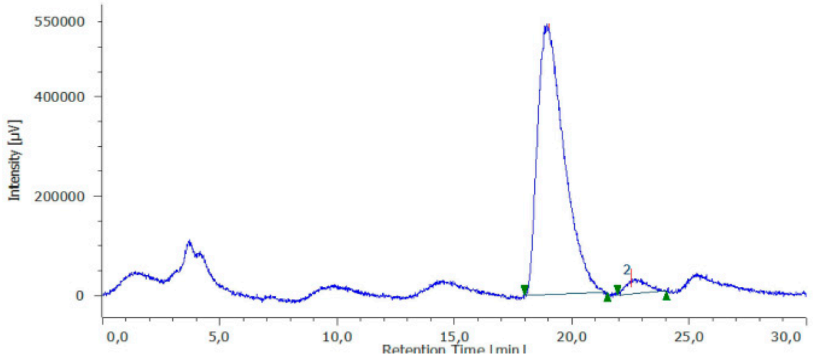

#	Peak Name	CH	tR [min]	Area [µV·sec]	Height [µV]	Area%
1	Unknown	5	19,027	42616905	554421	96,107
2	Unknown	5	22,547	1726115	31963	3,893

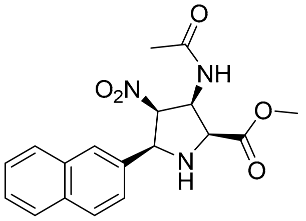

endo-**9ab**

Obtained using $AgClO_4$·(R_a,S,S)-**15**

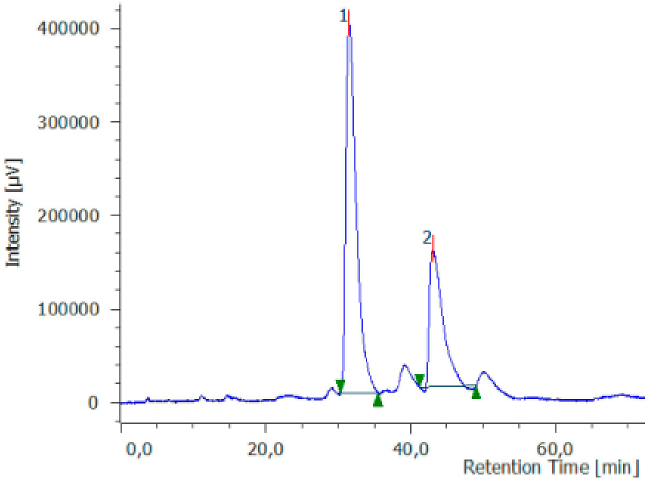

#	Peak Name	CH	tR [min]	Area [µV·sec]	Height [µV]	Area%
1	Unknown	11	31,507	39040649	395906	67,239
2	Unknown	11	43,120	19021771	146546	32,761

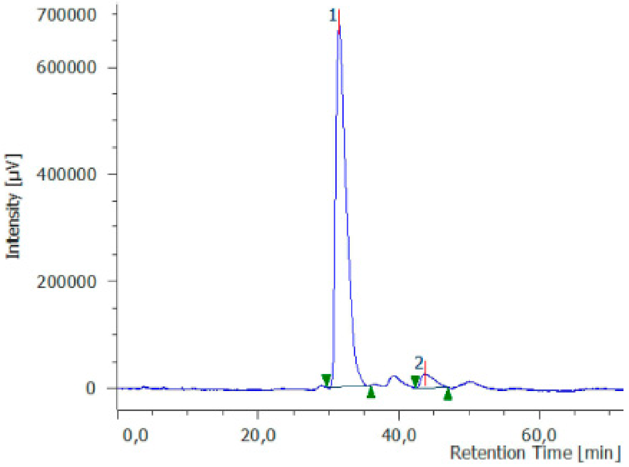

#	Peak Name	CH	tR [min]	Area [µV·sec]	Height [µV]	Area%
1	Unknown	11	31,453	70529994	681252	95,247
2	Unknown	11	43,693	3519454	27033	4,753

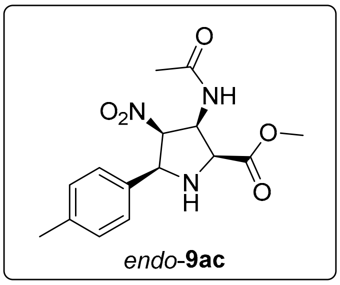

Obtained using $AgClO_4$·(R_a,S,S)-**15**

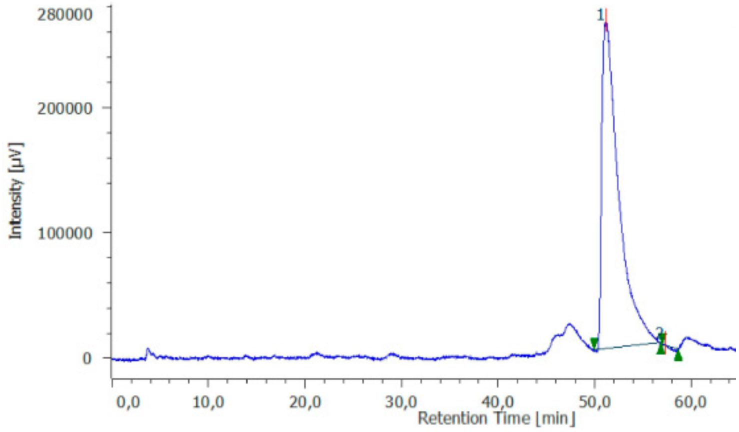

#	Peak Name	CH	tR [min]	Area [µV·sec]	Height [µV]	Area%
1	Unknown	11	50,853	27357362	240746	64,348
2	Unknown	11	56,853	15157332	121692	35,652

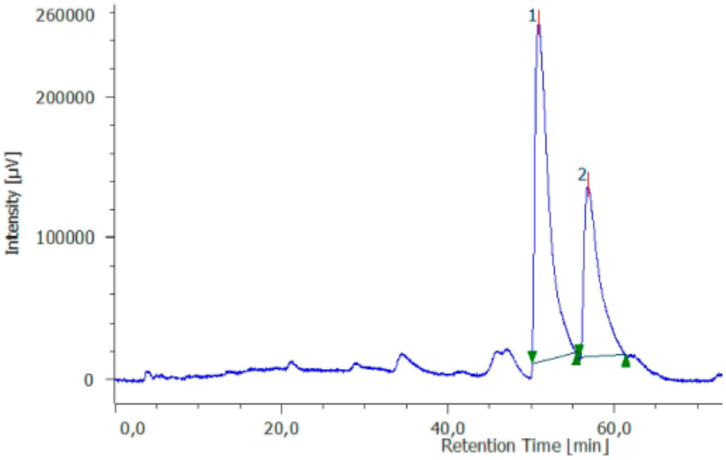

#	Peak Name	CH	tR [min]	Area [µV·sec]	Height [µV]	Area%
1	Unknown	11	51,187	31715251	260973	99,961
2	Unknown	11	57,280	12311	1899	0,039

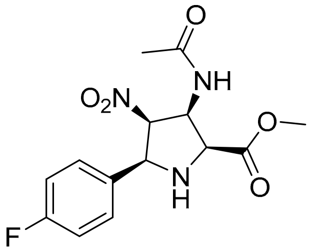

endo-**9ad**

Obtained using $AgClO_4$·(R_a,*S*,*S*)-**15**

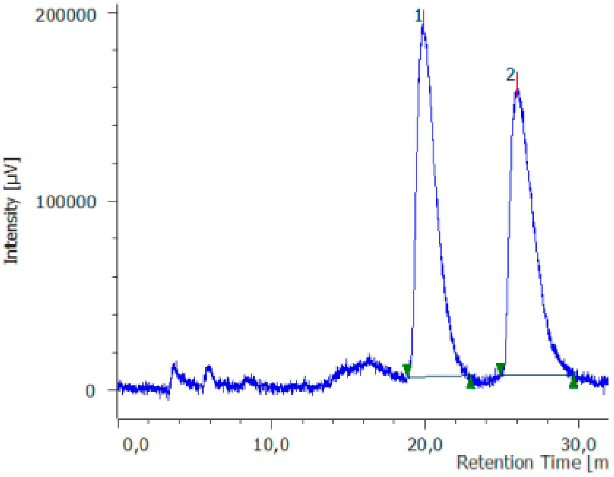

#	Peak Name	CH	tR [min]	Area [µV·sec]	Height [µV]	Area%
1	Unknown	5	19,947	16539196	187009	50,804
2	Unknown	5	26,000	16015602	153229	49,196

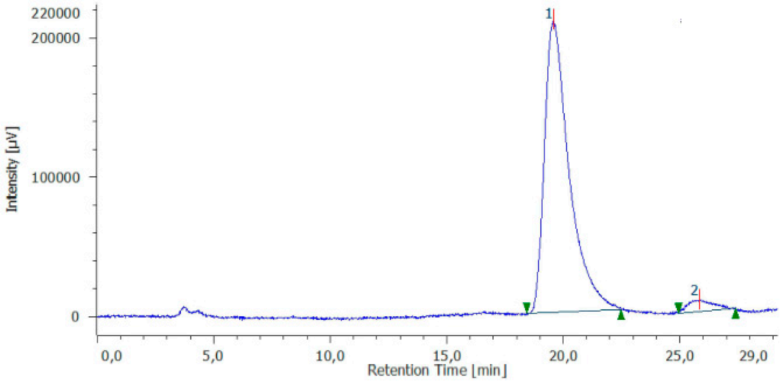

#	Peak Name	CH	tR [min]	Area [µV·sec]	Height [µV]	Area%
1	Unknown	11	19,573	15396861	210334	96,085
2	Unknown	11	25,813	627351	9037	3,915

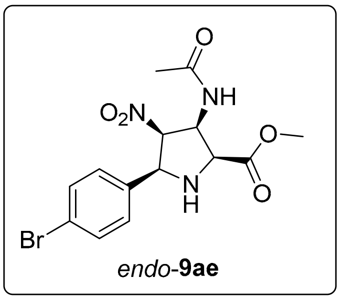

Obtained using $AgClO_4$·(R_a,*S*,*S*)-**15**

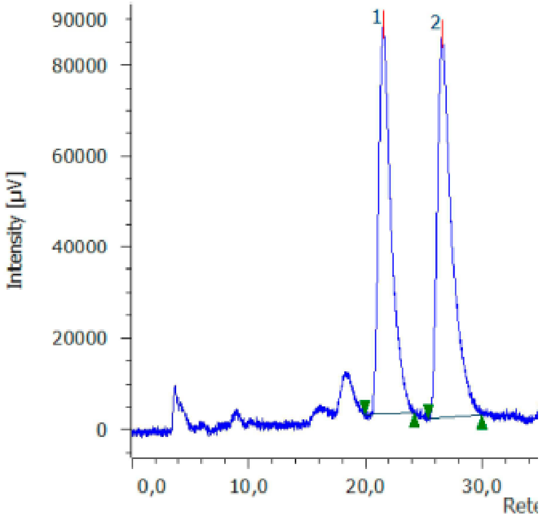

#	Peak Name	CH	tR [min]	Area [µV·sec]	Height [µV]	Area%
1	Unknown	11	21,520	6357024	85456	46,391
2	Unknown	11	26,533	7346071	84203	53,609

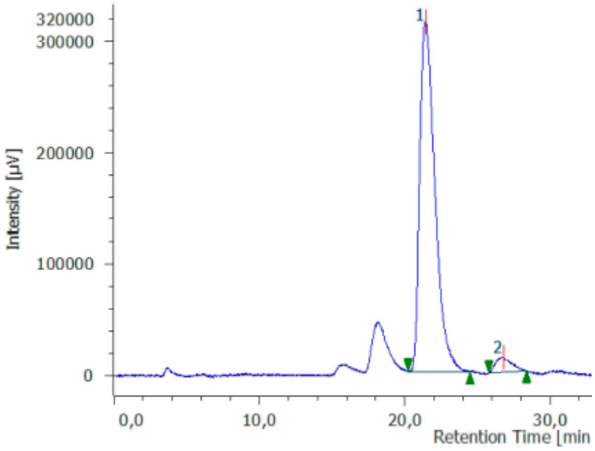

#	Peak Name	CH	tR [min]	Area [µV·sec]	Height [µV]	Area%
1	Unknown	11	21,440	22792529	313898	95,594
2	Unknown	11	26,800	1050547	13450	4,406

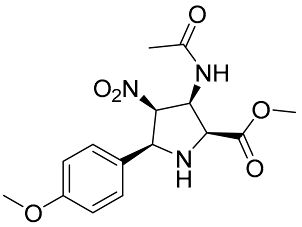

endo-**9af**

Obtained using $AgClO_4$·(R_a,S,S)-**15**

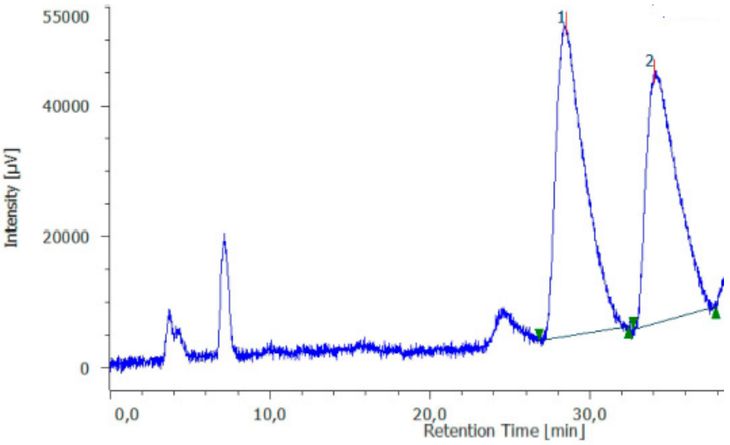

#	Peak Name	CH	tR [min]	Area [µV·sec]	Height [µV]	Area%
1	Unknown	11	28,533	5937906	47665	53,577
2	Unknown	11	34,067	5144939	38521	46,423

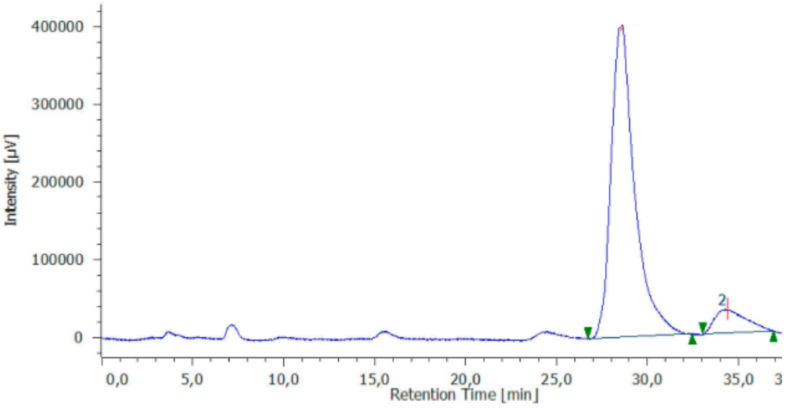

#	Peak Name	CH	tR [min]	Area [µV·sec]	Height [µV]	Area%
1	Unknown	11	12,560	15554140	336842	69,895
2	Unknown	11	20,173	6699291	107587	30,105

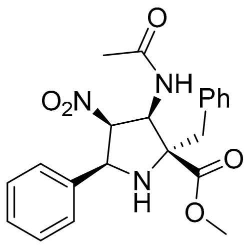

endo-**9ag**

Obtained using $AgClO_4$·(R_a,*S*,*S*)-**15**

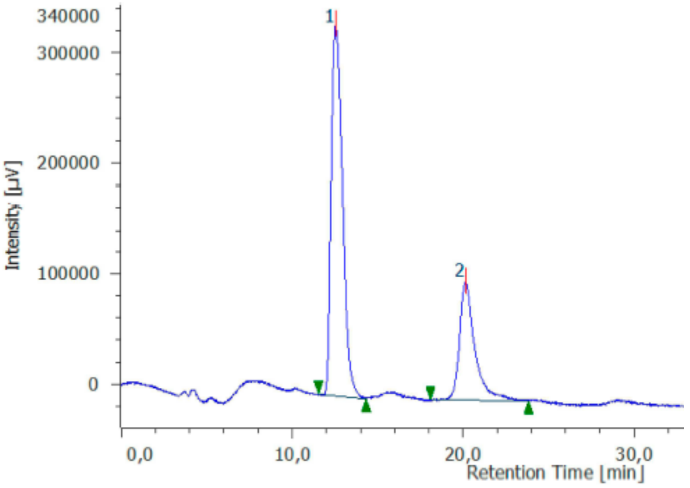

#	Peak Name	CH	tR [min]	Area [µV·sec]	Height [µV]	Area%
1	Unknown	11	12,560	15554140	336842	69,895
2	Unknown	11	20,173	6699291	107587	30,105

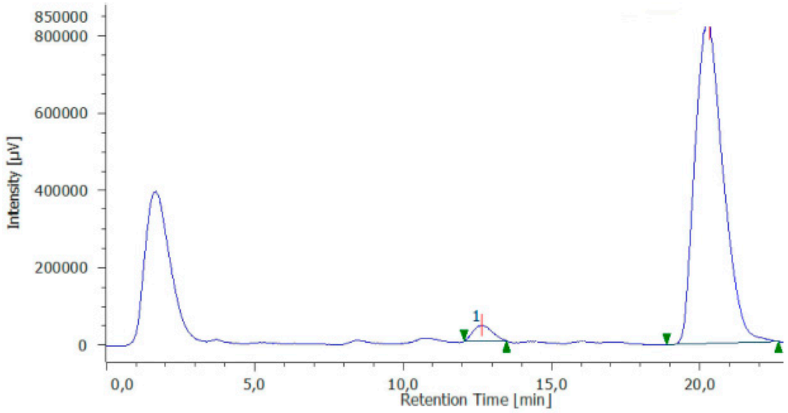

#	Peak Name	CH	tR [min]	Area [µV·sec]	Height [µV]	Area%
1	Unknown	11	12,640	1778185	40187	3,147
2	Unknown	11	20,333	54723891	811737	96,853

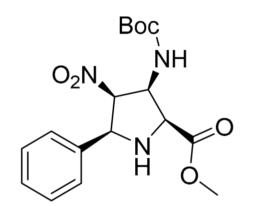

endo-**9ba**

Obtained using $AgClO_4$·(R_a,S,S)-**15**

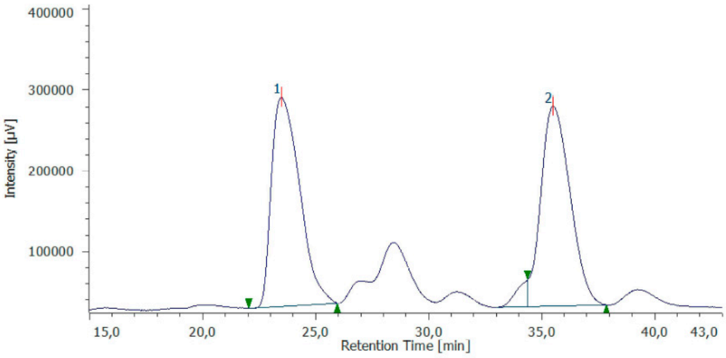

#	Peak Name	CH	tR [min]	Area [µV·sec]	Height [µV]	Area%	Height%	Quantity	NTP	Resolution	Symmetry Factor	Warning
1	Unknown	5	23,493	22527182	259396	50,179	51,052	N/A	1575	5,046	1,662	
2	Unknown	5	35,507	22366555	248703	49,821	48,948	N/A	3482	N/A	N/A	

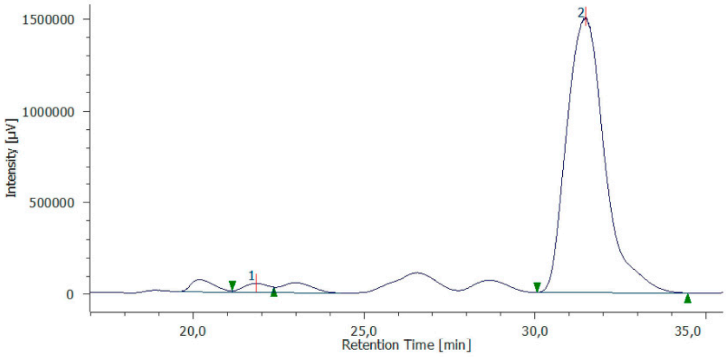

#	Peak Name	CH	tR [min]	Area [µV·sec]	Height [µV]	Area%	Height%	Quantity	NTP	Resolution	Symmetry Factor	Warning
1	Unknown	5	21,840	2463709	48543	2,089	3,125	N/A	N/A	N/A	N/A	
2	Unknown	5	31,480	115484522	1504902	97,911	96,875	N/A	4013	N/A	1,321	

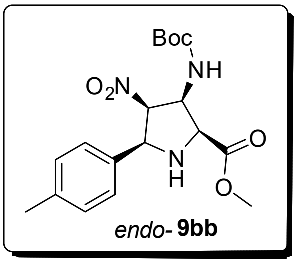

Obtained using $AgClO_4$·(R_a,S,S)-**15**

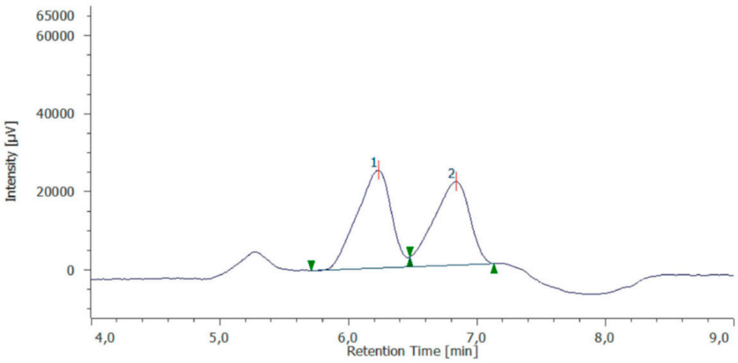

#	Peak Name	CH	tR [min]	Area [µV·sec]	Height [µV]	Area%	Height%	Quantity	NTP	Resolution	Symmetry Factor	Warning
1	Unknown	9	6,230	463261	25022	52,765	54,048	N/A	2445	1,174	N/A	
2	Unknown	9	6,837	414706	21274	47,235	45,952	N/A	2641	N/A	N/A	

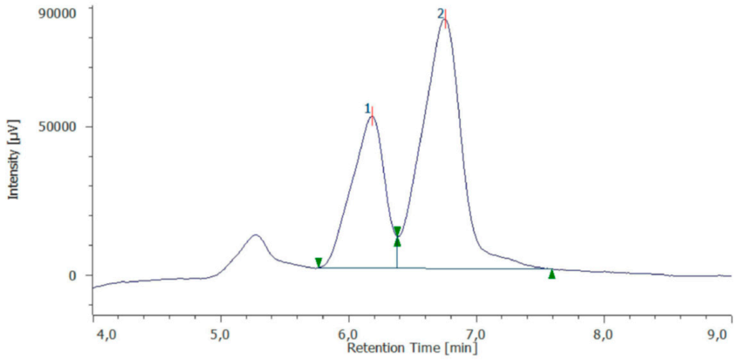

#	Peak Name	CH	tR [min]	Area [µV·sec]	Height [µV]	Area%	Height%	Quantity	NTP	Resolution	Symmetry Factor	Warning
1	Unknown	5	6,183	918552	51099	33,614	37,857	N/A	2502	1,086	N/A	
2	Unknown	5	6,753	1814084	83880	66,386	62,143	N/A	2342	N/A	N/A	

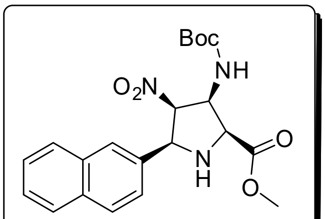

endo- **9bc**

Obtained using $AgClO_4$·(R_a,*S*,*S*)-**15**

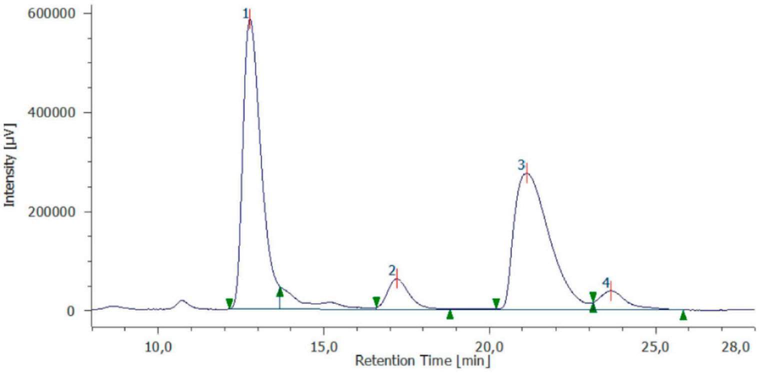

#	Peak Name	CH	tR [min]	Area [μV·sec]	Height [μV]	Area%	Height%	Quantity	NTP	Resolution	Symmetry Factor	Warning
1	Unknown	9	12,770	22278570	584788	46,999	60,999	N/A	2659	4,113	N/A	
2	Unknown	9	17,207	2814507	61504	5,937	6,415	N/A	3447	2,509	N/A	
3	Unknown	9	21,107	20096329	274347	42,395	28,617	N/A	1886	1,482	N/A	
4	Unknown	9	23,640	2213192	38044	4,669	3,968	N/A	4061	N/A	N/A	

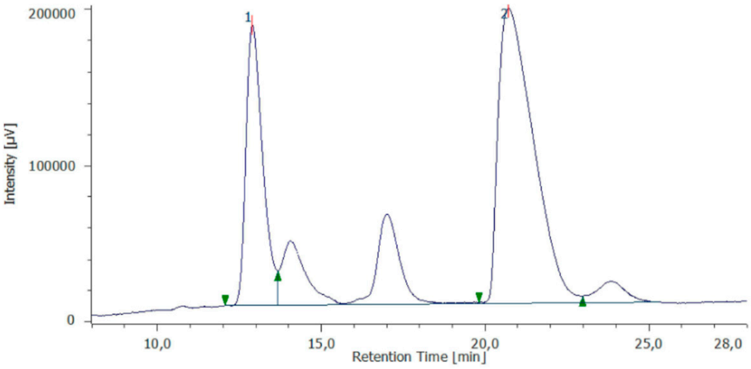

#	Peak Name	CH	tR [min]	Area [μV·sec]	Height [μV]	Area%	Height%	Quantity	NTP	Resolution	Symmetry Factor	Warning
1	Unknown	9	12,893	6393754	179191	31,164	48,684	N/A	3114	5,285	N/A	
2	Unknown	9	20,713	14122429	188880	68,836	51,316	N/A	1645	N/A	2,144	

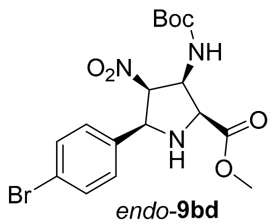

endo-**9bd**

Obtained using $AgClO_4$·(R_a,*S*,*S*)-**15**

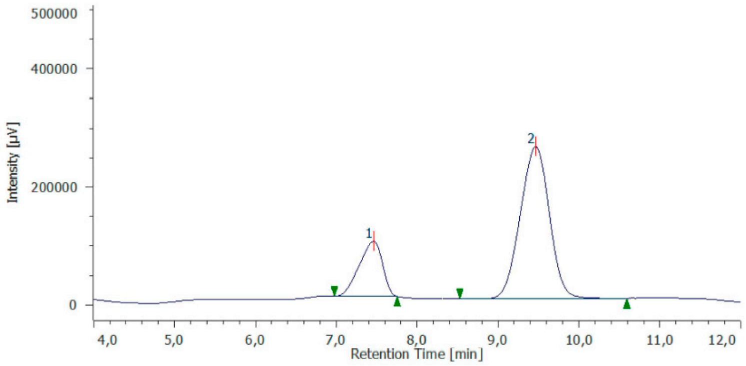

#	Peak Name	CH	tR [min]	Area [µV·sec]	Height [µV]	Area%	Height%	Quantity	NTP	Resolution	Symmetry Factor	Warning
1	Unknown	5	7,460	1874974	93175	22,557	26,521	N/A	2890	3,290	0,810	
2	Unknown	5	9,463	6437310	258149	77,443	73,479	N/A	3231	N/A	0,974	

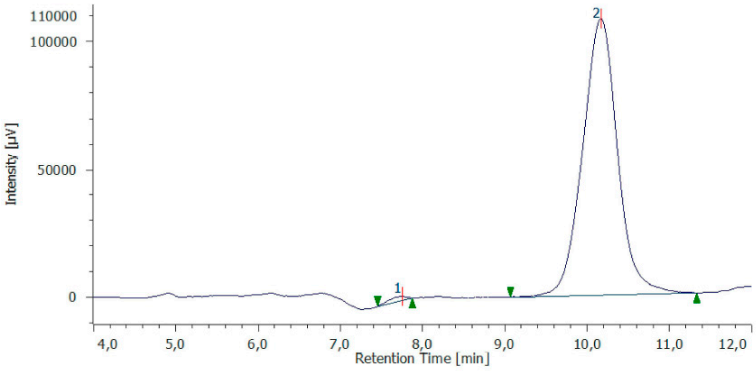

#	Peak Name	CH	tR [min]	Area [µV·sec]	Height [µV]	Area%	Height%	Quantity	NTP	Resolution	Symmetry Factor	Warning
1	Unknown	5	7,750	28229	1632	0,876	1,486	N/A	4538	3,995	0,705	
2	Unknown	5	10,167	3193503	108204	99,124	98,514	N/A	2918	N/A	1,030	

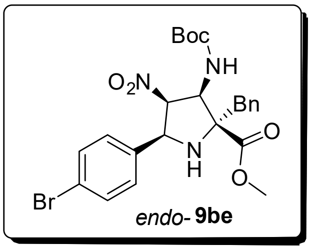

Obtained using AgClO4·(R_a,S,S)-**15**

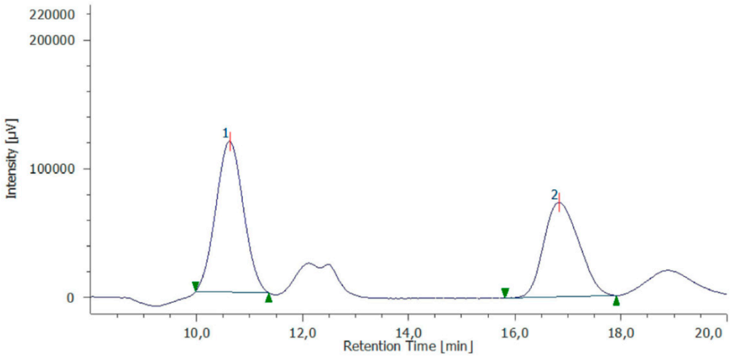

#	Peak Name	CH	tR [min]	Area [µV·sec]	Height [µV]	Area%	Height%	Quantity	NTP	Resolution	Symmetry Factor	Warning
1	Unknown	5	10,623	4223982	117060	55,603	61,508	N/A	1924	5,572	1,032	
2	Unknown	5	16,827	3372752	73255	44,397	38,492	N/A	2836	N/A	1,188	

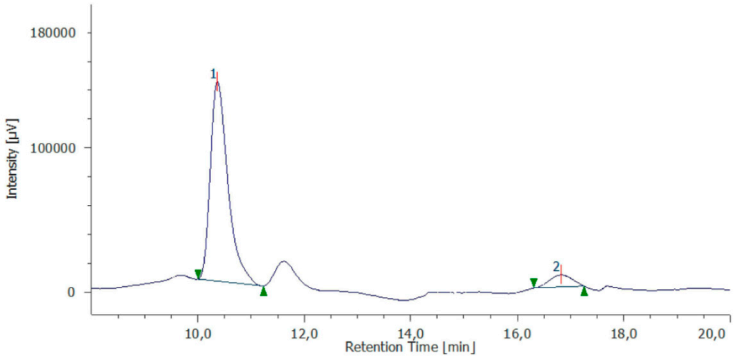

#	Peak Name	CH	tR [min]	Area [µV·sec]	Height [µV]	Area%	Height%	Quantity	NTP	Resolution	Symmetry Factor	Warning
1	Unknown	5	10,363	3172644	138345	92,685	94,212	N/A	5097	9,202	1,547	
2	Unknown	5	16,813	250412	8500	7,315	5,788	N/A	6646	N/A	0,968	
